# pH effects on plant calcium fluxes: lessons from acidification-mediated calcium elevation induced by the γ-glutamyl-leucine dipeptide identified from *Phytophthora infestans*

**DOI:** 10.1038/s41598-019-41276-0

**Published:** 2019-03-18

**Authors:** Lore Westphal, Nadine Strehmel, Lennart Eschen-Lippold, Nicole Bauer, Bernhard Westermann, Sabine Rosahl, Dierk Scheel, Justin Lee

**Affiliations:** 1 Department of Stress and Developmental Biology, Leibniz Institute of Plant Biochemistry (IPB), Halle (Saale), Germany; 2 Department of Bioorganic Chemistry, IPB, Halle (Saale), Germany

## Abstract

Cytosolic Ca^2+^ ([Ca^2+^]_cyt_) elevation is an early signaling response upon exposure to pathogen-derived molecules (so-called microbe-associated molecular patterns, MAMPs) and has been successfully used as a quantitative read-out in genetic screens to identify MAMP receptors or their associated components. Here, we isolated and identified by mass spectrometry the dipeptide γ-Glu-Leu as a component of a *Phytophthora infestans* mycelium extract that induces [Ca^2+^]_cyt_ elevation. Treatment of Arabidopsis seedlings with synthetic γ-Glu-Leu revealed stimulatory effects on defense signaling, including a weak enhancement of the expression of some MAMP-inducible genes or affecting the refractory period to a second MAMP elicitation. However, γ-Glu-Leu is not a classical MAMP since pH adjustment abolished these activities and importantly, the observed effects of γ-Glu-Leu could be recapitulated by mimicking extracellular acidification. Thus, although γ-Glu-Leu can act as a direct agonist of calcium sensing receptors in animal systems, the Ca^2+^-mobilizing activity in plants reported here is due to acidification. Low pH also shapes the Ca^2+^ signature of well-studied MAMPs (e.g. flg22) or excitatory amino acids such as glutamate. Overall, this work serves as a cautionary reminder that in defense signaling studies where Ca^2+^ flux measurements are concerned, it is important to monitor and consider the effects of pH.

## Introduction

Plants possess complex defense systems to prevent invasion by potentially harmful microorganisms. Essential for the initiation of defense is the detection of the microbes through binding of so-called microbe-associated molecular patterns (MAMPs) or molecules derived from damaged plant tissue (DAMP) to specific pattern recognition receptors (PRRs). Examples for pairs of MAMP/PRR identified in Arabidopsis are flagellin (epitope flg22)/FLAGELLIN SENSITIVE2 (FLS2)^1^, elongation factor Tu (EF-Tu, epitope elf18)/EF-TU RECEPTOR (EFR)^2,3^, as well as chitin/CHITIN ELICITOR RECEPTOR KINASE1 (CERK1)^4–6^ and LYSM RECEPTOR KINASE5 (LYK5)^7^. The DAMP AtPep1 is recognized by PEP RECEPTOR1/2 (PEPR1/2)^8–10^. Sensing of MAMPs/DAMPs by the PRRs activates a multicomponent signaling network. Signal transduction includes early physiological changes such as an increase in cytosolic Ca^2+^ and the production of reactive oxygen species (ROS), and employs phosphorylation pathways involving mitogen-activated protein kinase (MAPKs) cascades and calcium-dependent protein kinases (CPKs). These signaling processes lead to a reprogramming of gene expression, changes in phytohormone levels and other cellular signaling pathways, and ultimately in increased synthesis as well as secretion of antimicrobial substances^11–13^. The sum of all these induced defense responses constitutes the plant immune response termed pattern-triggered immunity (PTI)^14,15^.

A change in cytosolic Ca^2+^ concentration ([Ca^2+^]_cyt_) is one of the earliest observable response to environmental alterations, where kinetic variations in the Ca^2+^ transients or oscillations are thought to encode information^16^. Such “Ca^2+^ signatures” are decoded by a number of plant decoding mechanisms; these include Ca^2+^ binding proteins such as calmodulins (CaM), calcineurin B-like (CBL) proteins and CPKs (reviewed in^17^). The PAMP-induced alterations in [Ca^2+^]_cyt_ are dependent on the corresponding PRRs^18^. Reciprocally, when [Ca^2+^]_cyt_ is used as a readout in a genetic screen for mutants in early PAMP responses, the predominant mutants are the PRRs themselves, components of the receptor complex^19,20^ or elements of membrane protein (i.e. receptor) ER/Golgi quality control pathways^21,22^. This finding suggests that [Ca^2+^]_cyt_ measurements may be exploited to screen for PRRs of orphan MAMPs/DAMPs and as proof-of-principle for this idea, the ATP receptor^23^ and a putative lipopolysaccharide (LPS) receptor^24^ have been isolated by such a strategy.

In addition to Ca^2+^ influx, MAMP treatment also induces movement of other ions such as K^+^ efflux and H^+^ influx across the plasma membrane^25^. The resulting reduction of extracellular H^+^ concentration leads to an apoplastic alkalinization response^26,27^. The corresponding cytosolic acidification is believed to have signaling functions intracellularly^28,29^. Similarly, a link between Ca^2+^ signaling and pH dynamics has been observed in studies on developmental processes like the growth of pollen tubes^30^ and roots^31–34^, as well as in treatment of seedlings with different stimuli like exogenous auxin^35^ or mechanical stimulation^36^. In Arabidopsis roots, for example, oscillating changes in [Ca^2+^]_cyt_, extracellular pH and ROS might act together to control growth of root hair tips as well as root gravitropism^32–34^. Recently, parallel pH and Ca^2+^fluorescent reporter-based imaging after application of ATP, 1-naphthaleneacetic acid or wounding to Arabidopsis seedlings demonstrated that [Ca^2+^]_cyt_ and pH dynamics act in concert^37^. Thus, intracellular calcium and pH signaling are tightly intertwined.

*Phytophthora* species are classified as *Peronosporomycetes* (*Oomycetes*), which are phylogenetically distinct from true fungi. In fact, they belong to the *Stramenopiles* that comprise diatoms and brown algae^38^. Several *Phytophthora* species cause devastating diseases on major crops, such as potato late blight (*P*. *infestans*), soybean root rot (*P*. *sojae*) and ramorum blight on trees (*P*. *ramorum*). *P*. *infestans*, the causative agent of potato late blight, colonizes only members of the Solanaceae family; it is not adapted to infect non-solanaceous plants. Interaction of the nonhost Arabidopsis with this pathogen is characterized by the formation of cell wall depositions at sites of attempted infection in the epidermal cell layer, and in case of successful invasion, by a hypersensitive response of the affected cells^39,40^. Initial recognition of *P*. *infestans*, or generally *Phytophthora*, by plants presumably involves conserved MAMP molecules but their identity has been elusive. While some *Phytophthora* defense-triggering MAMPs (reviewed in^15,41,42^) are known, reports on *Phytophthora* MAMPs that elicit immunity in Arabidopsis are rare. The Cellulose-Binding Elicitor Lectin (CBEL) from *P*. *parasitica* var. *nicotianae* is a non-enzymatic cell wall glycoprotein with two cellulose-binding domains and lectin-like activities^43,44^. CBEL not only activates defense responses in the host tobacco^44^, but also in the nonhost Arabidopsis^45^, where CBEL perception might occur indirectly via alterations of the cellulose status in the plant cell wall^46^. Necrosis- and ethylene-inducing peptide1-like proteins (NLPs) are microbial effectors secreted by a wide range of bacteria, fungi and *Peronosporomycetes*^47,48^ and it was initially unclear if plant defense is receptor-mediated or through cellular damage from tissue necrosis. Subsequently, a conserved 20-mer amino acid sequence (nlp20) present in both cytotoxic and non-cytotoxic NLPs proved to be sufficient to trigger defense responses in a variety of *Brassicaceae*^49^. In this case, recognition of nlp20 occurs through the PRR complex consisting of the receptor-like protein RLP23, the receptor-like kinase SOBIR1 (SUPPRESSOR OF BAK1-INTERACTING RECEPTOR KINASE1 (BIR1)) and BAK1^50,51^.

The initial aim of our study was to isolate MAMPs from *P*. *infestans* and initiate a genetic screen based on [Ca^2+^]_cyt_ measurements to identify the corresponding PRR(s). Here, we isolated and identified the dipeptide γ-Glu-Leu as a component of a *P*. *infestans* mycelium extract that induces [Ca^2+^]_cyt_ elevation. Treatment of Arabidopsis seedlings with γ-Glu-Leu showed that it could promote some defense responses to MAMPs. However, pH adjustment of the dipeptide solution abolished most of the effects, highlighting the impact of acidification on defense-related signaling, a largely overlooked and underestimated phenomenon.

## Results

### Ca^2+^ flux inducing *P*. *infestans* mycelium extract contains the dipeptide γ-Glu-Leu

**Figure 1:**
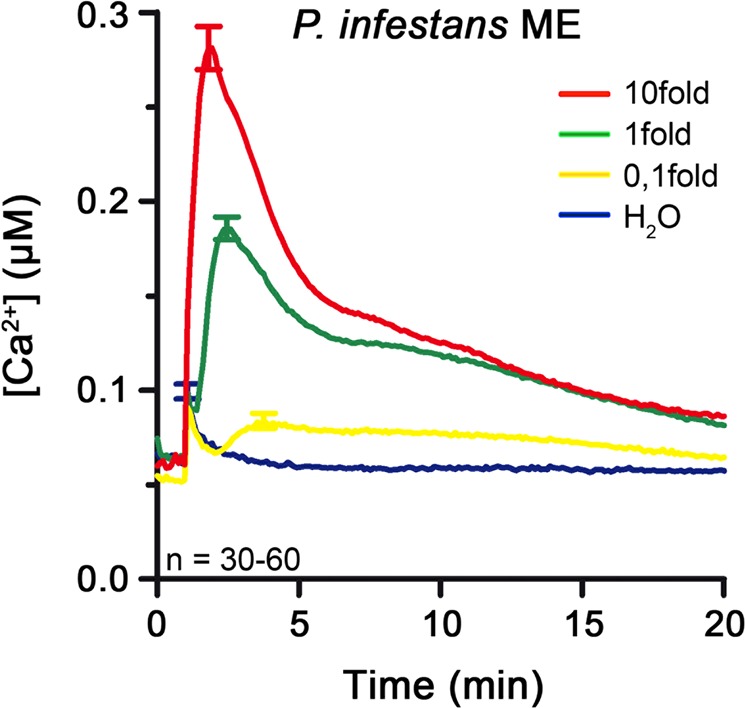
*P*. *infestans* mycelium extract elicits Ca^2+^ flux in Arabidopsis pMAQ2 in a concentration-dependent manner. Seedlings were treated with water, 0.1, 1 and 10fold *P*. *infestans* mycelium extract, respectively, and [Ca^2+^]_cyt_ levels were estimated (at least 3 independent experiments per concentration). Error bars represent standard error of the mean. ME = mycelium extract.

To isolate *P*. *infestans* MAMPs that trigger immune responses in its nonhost Arabidopsis, we isolated elicitor(s) extracted from liquid culture-grown mycelium following a protocol modified from Monjil *et al*.^52^. The authors reported that their extract caused growth retardation in Arabidopsis seedlings and induced an oxidative burst, hypersensitive response and defense gene expression when infiltrated into leaves, but so far there is no information available on the active component(s). When we challenged aequorin-expressing Arabidopsis (pMAQ2) seedlings with the crude *P*. *infestans* mycelium extract, we observed a transient increase in [Ca^2+^]_cyt_ with a peak height of 186 ± 6 nM followed by a prolonged decline to basal level, which lasted more than 15 minutes (Fig. 1). A 10-fold diluted or concentrated extract triggered a similar [Ca^2+^]_cyt_ profile but with correspondingly lower or higher overall amplitude and peaks (84 ± 4 nM or 281 ± 11 nM, respectively) (Fig. 1).

**Figure 2:**
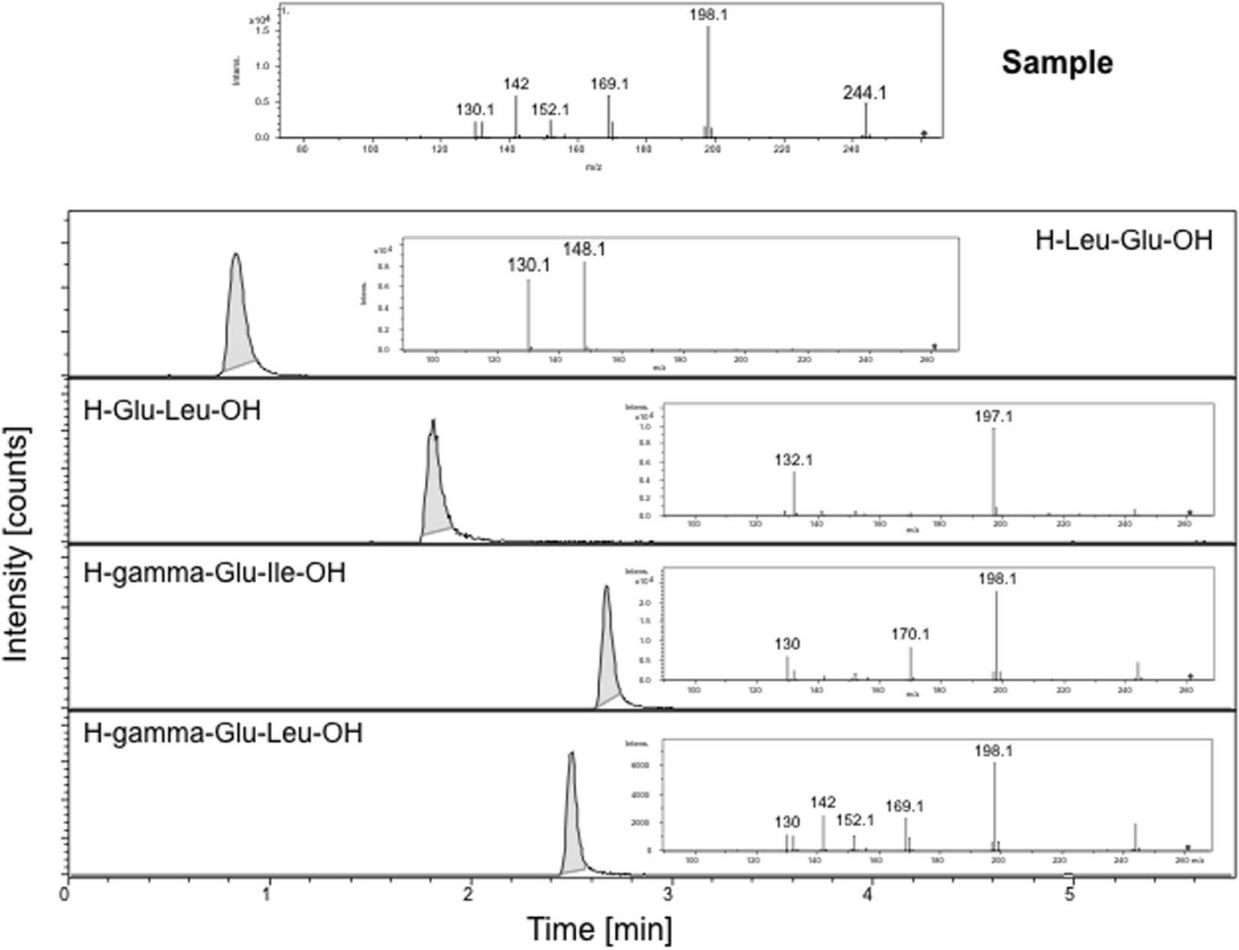
CID-MS Spectra of isomers matching the elemental composition C_11_H_20_N_2_O_5_^+^. The *P*. *infestans* mycelium extract peak was annotated as gamma-glutamyl-leucine according to retention time and mass spectral features. The identity was confirmed using an authenticated reference substance.

In pilot fractionation experiments, the [Ca^2+^]_cyt_ elevation-inducing activity was found in the fractions eluted with water from the C_18_ silica solid phase extraction cartridge. Thus, the active component(s) do not bind strongly to the octadecyl carbon chains of the reverse-phase columns and is/are likely hydrophilic in nature. To identify putative elicitor(s), this water-elutable mycelium extract fraction was subjected to an untargeted mass spectrometry (MS) analysis. Samples were measured in positive (ESI(+)) and negative (ESI(−)) ionization mode and evaluated separately. Five compounds were extracted from the ESI(+) profiles and four compounds from the ESI(−) profiles (Fig. S1). In total, five compounds (including citric acid, isocitric acid, trigonelline, and tyrosine) were annotated based on collision-induced dissociation (CID) experiments and H/D exchange experiments and confirmed by the use of authenticated reference substances (Table 1). Compounds exhibiting the elemental composition C_14_H_18_N_2_O_6_, C_16_H_22_O_10_, C_9_H_17_NO_4_, C_11_H_20_N_2_O_5_, and C_9_H_10_O_5_ were also found in the metabolite profiles, but could not be assigned to a chemical structure yet. C_11_H_20_N_2_O_5_^+^ was annotated as γ-glutamyl-leucine (γ-Glu-Leu), because it exhibits characteristic MS fragments at *m/z* 244.11 (C_11_H_18_NO_5_^+^, [M + H-NH_3_]^+^), 215.14 (C_10_H_19_N_2_O_3_^+^, [M + H-HCO_2_]^+^), 198.11 (C_10_H_16_NO_3_^+^, [M + H-HCO_2_-NH_3_]^+^), 170.12 (C_9_H_16_NO_2_^+^, [M + H-NH_3_-CO-HCO_2_]), 132.1 (C_6_H_14_NO_2_^+^, [Leu + H]^+^), 130.05 (C_5_H_8_NO_3_^+^, [Glu + H-H_2_O]^+^) resulting from neutral losses and alpha cleavages (Table 1). For further validation, Leu-Glu, Glu-Leu, and γ-Glu-Ile were either purchased (when commercially available) or synthesized in-house. Compared to γ-Glu-Leu, these isomers showed different fragmentation patterns and retention times and were therefore excluded (Fig. 2). Using calibration curves of the authentic compound, the concentration of γ-Glu-Leu in the *P*. *infestans* mycelium extract was estimated to be 110–190 nM (Fig. S2).

**Table 1:** Analytical data of detected compounds.

ID	Compound	VL	ESI	t_r_ [min]	measured *m/z*	mSigma	Elemental composition	precursor CE [eV]	observed fragment ions upon CID	t_r_ [min]	measured *m/z*	# exchang. protons
1	Citric acid	S	neg	0.5	191.0208	4.9	C_6_H_7_O_7_^−^	[M]^−^, 10	**191** (9, C_6_H_7_O_7_^−^), 173.01 (2, C_6_H_5_O_6_^−^), 147.03 (1, C_5_H_7_O_5_^−^), 129.02 (2, C_5_H_5_O_4_^−^), 111.01 (100, C_5_H_3_O_3_^−^)	0.5	194.0395	4
2	Isocitric acid	S	neg	0.6	191.02	6.3	C_6_H_7_O_7_^−^	[M]^−^, 10	**191** (8, C_6_H_7_O_7_^−^), 173.01 (2, C_6_H_5_O_6_^−^), 147.03 (1, C_5_H_7_O_5_^−^), 129.02 (7, C_5_H_5_O_4_^−^), 111.01 (100, C_5_H_3_O_3_^−^)	0.7	194.0392	4
3	Unknown		neg	0.9	309.1122	24.8	C_14_H_17_N_2_O_6_^−^	[M]^−^, 20	**309** (4, C_14_H_17_N_2_O_6_^−^), 146.08 (100, C_6_H_12_NO_3_^−^)	1.0	313.1368	5
4	Unknown		neg	2.8	373.1099	14.3	C_16_H_21_O_10_^−^	[M]^−^, 10	**373** (56, C_16_H_21_O_10_^−^), 355.1 (2, C_16_H_19_O_9_^−^), 313.09 (3, C_14_H_17_O_8_^−^), 197.04 (2, C_9_H_9_O_5_^−^), 175.06 (100, C_7_H_11_O_5_^−^), 157.05 (1, C_7_H_9_O_4_^−^), 115.04 (2, C_5_H_7_O_3_^−^)	3.0	377.1361	5
1	Trigonelline	S	pos	0.5	138.0551	8.1	C_7_H_8_NO_2_^+^	[M]^+^, 30	**138** (100, C_7_H_8_NO_2_^+^), 136.04 (8, C_7_H_6_NO_2_^+^)	0.6	139.0622	0
2	Unknown		pos	0.6	204.1228	6.6	C_9_H_18_NO_4_^+^	[M]^+^, 20	**204** (34, C_9_H_18_NO_4_^+^), 162.11 (2, C_7_H_16_NO_3_^+^), 144.1 (100, C_7_H_14_NO_2_^+^), 129.08 (12, C_6_H_11_NO_2_^+.^)	0.5	205.1299	0
3	Tyrosine	S	pos	0.7	182.0806	2.5	C_9_H_12_NO_3_^+^	[M]^+^, 10	**182** (10, C_9_H_12_NO_3_^+^), 165.05 (67, C_9_H_9_O_3_^+^), 147.04 (25, C_9_H_7_O_2_^+^), 136.08 (100, C_8_H_10_NO^+^), 123.04 (20, C_7_H_7_O_2_^+^), 119.05 (9, C_8_H_7_O^+^)	0.7	187.1126	4
4	gamma Glu-Leu	S	pos	2.6	261.1428	1.9	C_11_H_21_N_2_O_5_^+^	[M]^+^, 10	**261** (100, C_11_H_21_N_2_O_5_^+^), 244.11 (50, C_11_H_18_NO_5_^+^), 215.14 (1, C_10_H_19_N_2_O_3_^+^), 198.11 (34, C_10_H_16_NO_3_^+^), 170.12 (1, C_9_H_16_NO_2_^+^), 132.1 (29, C_6_H_14_NO_2_^+^), 130.05 (4, C_5_H_8_NO_3_^+^)	2.8	267.1811	5
5	Unknown		pos	2.8	199.0565	11.5	C_9_H_11_O_5_^+^	[M]^+^, 10	**199** (100, C_9_H_11_O_5_^+^), 181.04 (2, C_9_H_9_O_4_^+^), 153.05 (1, C_8_H_9_O_3_^+^)	3.0	202.0759	2

VL: verification level; S: standard; t_r_: retention time; ESI: electrospray ionization mode, observed fragment ions upon CID *m/z* (rel. int. [%], elemental composition), precursor ion is marked in bold.

### γ-Glu-Leu induces Ca^2+^ flux and desensitizes cells for elicitation with *P*. *infestans* mycelium extract and different MAMPs

**Figure 3:**
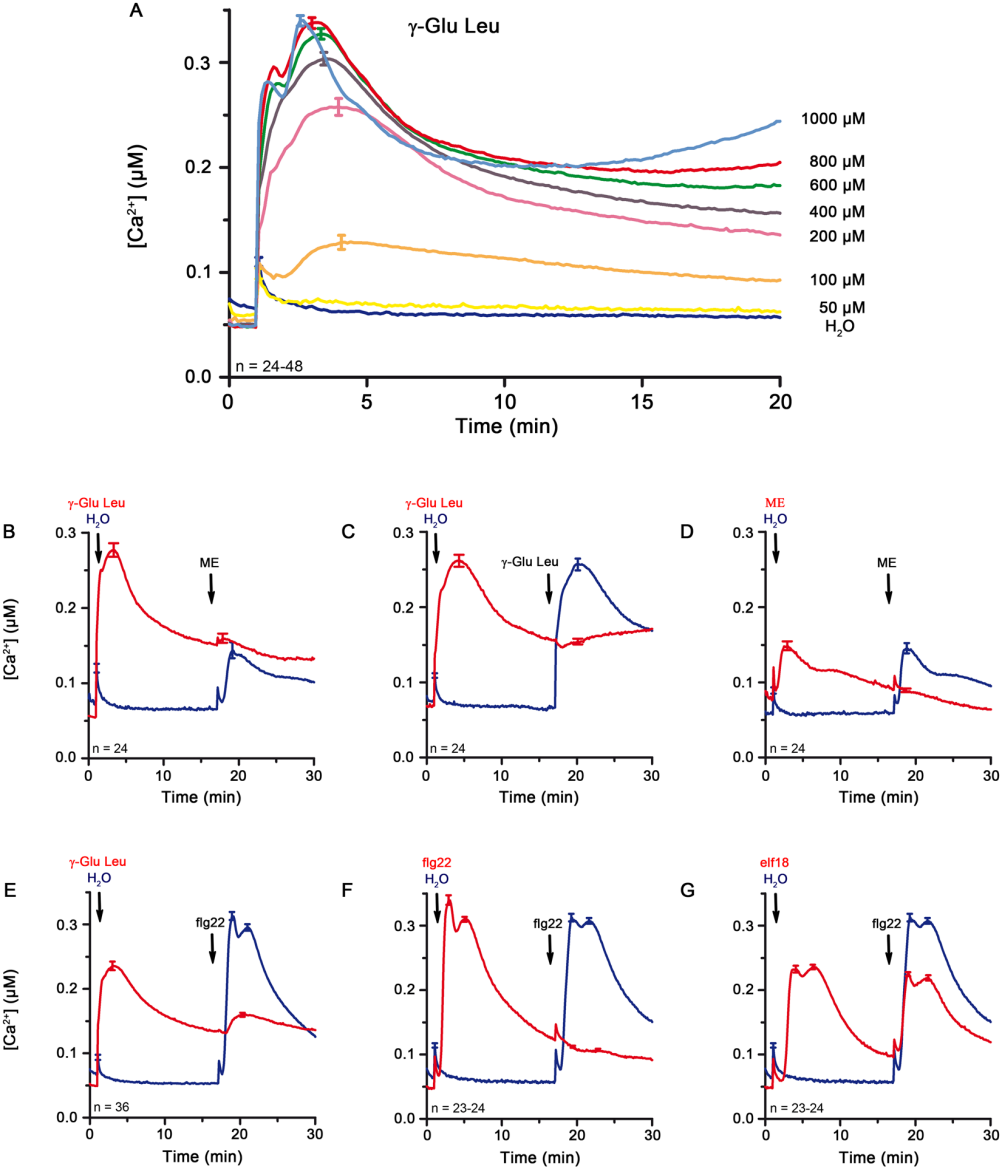
γ-Glu-Leu induces Ca^2+^ flux in Arabidopsis pMAQ2 in a concentration-dependent manner and desensitizes seedlings for subsequent treatments with *P*. *infestans* mycelium extract or flg22. (**A**) Seedlings were treated with water and aqueous solutions containing different concentrations of γ-Glu-Leu, respectively (two to four independent experiments per concentration). (**B**–**G**) Elicitation of seedlings with water or aqueous solutions of 500 µM γ-Glu-Leu (**B**,**C**,**E**), 1-fold mycelium extract (**D**), 1 µM flg22 (**F**) or 1 µM elf18 (**G**) for approximately 15 min was followed by the application of a second stimulus (**B**,**D**: 1-fold mycelium extract, C: 500 µM γ-Glu-Leu, E, F, G: 1 µM flg22). Experiments were performed two to three times and the curves display the pooled [Ca^2+^]_cyt_ data from the repeated experiments. Error bars represent standard error of the mean. ME = *P*. *infestans* mycelium extract.

Several γ-glutamyl peptides have been discovered to be *kokumi* (taste-enhancing) substances and to induce a rise in intracellular [Ca^2+^] in taste receptor cells in the lingual tissues of mice^53^. These γ-glutamyl *kokumi* peptides appear to directly engage calcium-sensing receptor (CaSR) in the taste buds^54^. Thus, among the identified components in the mycelium extract, γ-Glu-Leu is a candidate Ca^2+^ flux-inducing compound and acts similarly in plants as an agonist of calcium channels. Alternatively, it may act as a MAMP to induce Ca^2+^ elevations. To test if the dipeptide γ-Glu-Leu is indeed the active component from the mycelium extract that induces the [Ca^2+^]_cyt_ increase, we applied synthetic γ-Glu-Leu (ranging from 50 to 1000 µM) to aequorin-expressing Arabidopsis seedlings. Treatment with 50 µM γ-Glu-Leu had no obvious effect on the basal level of [Ca^2+^]_cyt_ represented by the water control, whereas all higher concentrations induced a transient elevation in Ca^2+^ flux with dose-dependent magnitudes between 129 ± 7 nM [Ca^2+^]_cyt_ for 100 µM and 340 ± 5 nM [Ca^2+^]_cyt_ for 1000 µM γ-Glu-Leu (Figs 3A and S3). Based on the peak [Ca^2+^]_cyt_ values in these experiments, the EC_50_ value of γ-Glu-Leu was estimated to be approximately 160 µM. The MAMP-induced Ca^2+^ signature reported by the aequorin system is typically a transient Ca^2+^ rise, followed by a return to basal resting levels. However, we observed that the rate of recovery to basal [Ca^2+^]_cyt_ level decelerated with increasing γ-Glu-Leu concentrations (Fig. 3A). In fact, for the 800 µM and 1000 µM γ-Glu-Leu treatments, a “second wave” of Ca^2+^ rise appears to be initiated at ~12 min (Fig. 3A). Taken together, γ-Glu-Leu is likely an active component within the *P*. *infestans* mycelium extract, which elicits Ca^2+^ elevations. If γ-Glu-Leu functions as a typical MAMP, the EC_50_ value of the Ca^2+^ induction would be indicative of a receptor-ligand interaction of low-to-moderate affinity.

Activated receptors are typically desensitized and unable to be re-activated within the so-called refractory period (for example, see Fig. 3F). This would be the scenario if γ-Glu-Leu engages the same receptor as *P*. *infestans* mycelium extract (ME). To test this, pMAQ2 seedlings were treated with a saturating concentration of γ-Glu-Leu (500 µM) for ~15 min prior to a second stimulation with ME. In comparison to the control (with water as pretreatment), γ-Glu-Leu severely reduced Ca^2+^ peaks obtained with a subsequent application of ME (Fig. 3B). Similar suppression of the second Ca^2+^ peak induction was seen with consecutive applications of γ-Glu-Leu (Fig. 3C) or ME (Fig. 3D). Surprisingly, while not as strong as the γ-Glu-Leu/ME combination, pre-stimulation with γ-Glu-Leu also suppressed the Ca^2+^ peaks elicited by subsequent application with flg22 (1 µM) (Fig. 3E). Similar observations were seen if the second treatment used was elf18 (1 µM), PEP (1 µM) or chitin (200 µg shrimp shell/ml) (Fig. S4A). This is reminiscent of the refractory period observed with consecutive applications of the same PAMP (e.g. flg22, Fig. 3F), which is not seen if two independent receptors are involved (e.g. elf18 treatment followed by flg22 as a second application, Fig. 3G). However, since it is unlikely that γ-Glu-Leu can act as an agonist for all these different receptors, another plausible explanation is that γ-Glu-Leu shares certain common signaling component(s) with these MAMPs/DAMPs to elicit the Ca^2+^ response. For instance, γ-Glu-Leu may act similarly to the *kokumi* substances and engage the same plant Ca^2+^ channels activated by the different MAMP/DAMPs. Thus, analogous to the taste-enhancing properties in animal systems, γ-Glu-Leu may have synergistic effects on plant defense.

### Simultaneous application of γ-Glu-Leu and flg22 modulates defense responses obtained with single treatments

**Figure 4:**
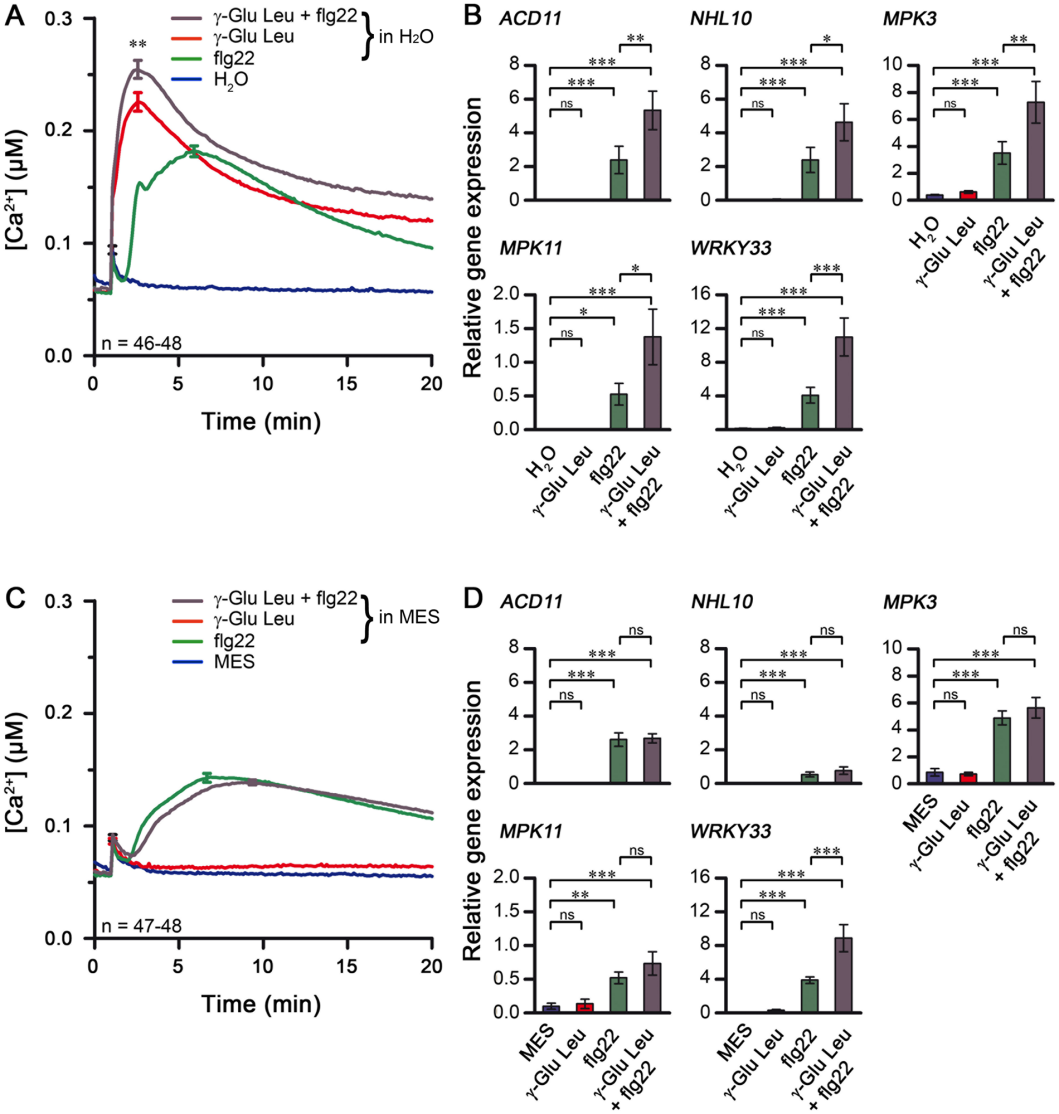
Co-treatment of Arabidopsis pMAQ2 with γ-Glu-Leu and flg22 enhances activation of Ca^2+^ flux and defense gene expression in comparison to the single treatments (**A**,**B**), but boosting effects are lost when elicitors are MES-buffered (**C**,**D**). (**A**) Seedlings were treated with water or an aqueous solution containing 10 nM flg22, 250 µM γ-Glu-Leu or a mixture of 10 nM flg22 and 250 µM γ-Glu-Leu. [Ca^2+^]_cyt_ curves are pooled from four independent experiments. Error bars represent standard error of the mean. **Significant difference (P < 0.01) according to Two-way ANOVA with Bonferroni post-test. (**B**) Pools of seedlings were elicited with water or an aqueous solution containing 10 nM flg22 and/or 500 µM γ-Glu-Leu for 1 hour. Expression of defense genes (relative to the reference gene *PP2AA3*) was determined in two independent experiments with 3–4 seedling pools per treatment and 2 technical replicates per pool. Combined data of both experiments were log-transformed prior to One-way ANOVA with Bonferroni multiple comparison tests for selected columns, as indicated. *,**,***Significant difference (P < 0.05, 0.01, 0.001, respectively), ns: not significant (**C**) Seedlings were treated with MES (pH 6) or 10 nM flg22, 250 µM γ-Glu-Leu or a mixture of 10 nM flg22 and 250 µM γ-Glu-Leu (in MES buffer, pH 6.0). Experiments were performed four times and [Ca^2+^]_cyt_ data were pooled. Error bars represent standard error of the mean. (**D**) Pools of seedlings were elicited with MES (pH 6.0) or a MES solution containing 10 nM flg22 and/or 500 µM γ-Glu-Leu (pH 6.0) for 1 hour. Gene expression data was pooled from two independent experiments and analyzed as described in B above.

The observations above suggest possible interplay between γ-Glu-Leu and MAMPs. As anticipated for situations resembling natural infections where there is simultaneous exposure to several MAMPs, combinatorial co-treatment with different MAMPs/DAMPs has been reported to confer additive, synergistic or reductive effects on defense responses in comparison to the single stresses^55–57^. We therefore tested whether a combination of γ-Glu-Leu and flg22 (as a representative MAMP) will affect Arabidopsis defense responses differently compared to single treatments. We compared the effect of single or combined stimuli on Ca^2+^ flux as well as relative transcript levels of several flg22-responsive defense genes. Simultaneous treatment with non-saturating concentrations of γ-Glu-Leu (250 µM) and flg22 (10 nM) induced a similar Ca^2+^ signature to that obtained with γ-Glu-Leu alone but with a significantly slightly higher amplitude of 255 ± 8 nM [Ca^2+^]_cyt_ (Fig. 4A).

To evaluate the effect on defense, we tested expression of twelve routinely analyzed flg22-responsive marker genes: *ACCELERATED CELL DEATH11-like* (*ACD11-like*), *CINNAMYL ALCOHOL DEHYDROGENASE5* (*CAD5*), *FLG22-INDUCED RECEPTOR-LIKE KINASE1* (*FRK1*), *MITOGEN-ACTIVATED PROTEIN KINASE3* (*MPK3*), *MITOGEN-ACTIVATED PROTEIN KINASE11* (*MPK11*), *NDR1/HIN1-LIKE10* (*NHL10*), *PHYTOALEXIN DEFICIENT3* (*PAD3*), *PHYTOCHELATIN SYNTHASE1* (*PCS1*), *PHOSPHATE-INDUCED1* (*PHI1*), *WRKY DNA-BINDING PROTEIN33* (*WRKY33*), *WRKY DNA-BINDING PROTEIN53* (*WRKY53*) and *ZINC FINGER PROTEIN12* (*ZAT12*)^58,59^. When applied alone, γ-Glu-Leu did not induce the expression of any of these genes even at the high concentration of 500 µM (Figs 4B and S5). However, compared to flg22 alone, co-treatment with 500 µM γ-Glu-Leu and 10 nM flg22 resulted in a significant increase in transcript levels of *ACD11-like*, *NHL10*, *MPK3*, *MPK11* and *WRKY33* (Fig. 4B) but not of the other genes (Fig. S5).

Taken together, γ-Glu-Leu confers subtle “additive” effects on flg22-induced Ca^2+^ flux and expression of a subset of defense genes. Since many of the analyzed genes are controlled by MAPK and/or CPK pathways^58,59^, the enhancement of gene expression may result from increased activities of these kinases. However, despite the enhanced [Ca^2+^]_cyt_, γ-Glu-Leu treatment did not raise basal or flg22-induced activity of CPK (using in-gel phosphorylation assays with histone as substrate, data not shown) or MAPK phosphorylation (as a proxy for MAPK activity, Fig. S6A). For the latter, γ-Glu-Leu weakly induces MPK6 phosphorylation but showed no further enhancement of MAPK activation when co-treated with flg22 (Fig. S6B). Since PAMPs only activate MAPKs transiently and to exclude the possibility that “the window” of any additive effect of the co-treatment was overlooked in the time points analyzed, we monitored an effect downstream of MPK6. For this, we checked the phosphorylation state of the MPK6 substrate, MVQ1, which can be visualized as a reduced electrophoretic mobility in SDS-PAGE after MAMP treatment^60^. Unlike flg22, neither γ-Glu-Leu alone, nor in combination with flg22 induced or further enhanced MVQ1 phospho-mobility shift, respectively (Fig. S6C). Hence, the boost in Ca^2+^ flux or gene expression using co-treatment is unlikely to be caused by enhanced MAPK or CPK kinase activities.

### pH alteration may constitute the observed biological activity of γ-Glu-Leu

**Figure 5:**
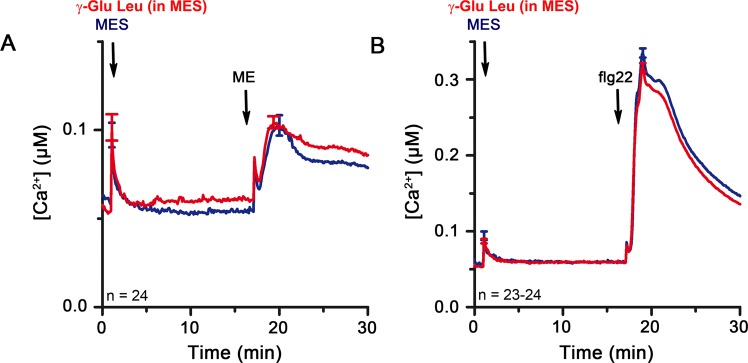
γ-Glu-Leu dissolved in MES buffer (pH 6.0) has no obvious effect on Ca^2+^ flux and the response to a second elicitor. (**A**,**B**) pMAQ2 seedlings were treated with MES (pH 6.0) or 500 µM γ-Glu-Leu (in MES buffer, pH 6.0) for approximately 15 min prior to the application of (**A**) 1-fold mycelium extract and (**B**) 1 µM flg22. [Ca^2+^]_cyt_ data of two independent experiments were pooled. Error bars represent standard error of the mean. ME = *P*. *infestans* mycelium extract.

Cytosolic acidification is known to weakly activate MAPKs^61^. It is, thus, plausible that γ-Glu-Leu, with its additional acidic side group of the Glu moiety, promotes apoplastic and subsequently cytosolic acidification. Indeed, aqueous γ-Glu-Leu solution has an acidic pH of 3.9. When γ-Glu-Leu was dissolved in MES-buffered solution (pH 6.0), it no longer induced a rise in [Ca^2+^]_cyt_ and the additive effects of flg22 co-treatment on Ca^2+^ flux (Fig. 4C), as well as on defense gene expression were also lost except for *WRKY33* (Fig. 4D). Similarly, the apparent “refractory period” conferred by γ-Glu-Leu pretreatment to subsequent elicitation with ME or MAMPs (Fig. 3B,E) was also abolished if γ-Glu-Leu was MES-buffered at pH 6.0 (Figs 5 and S4B).

**Figure 6:**
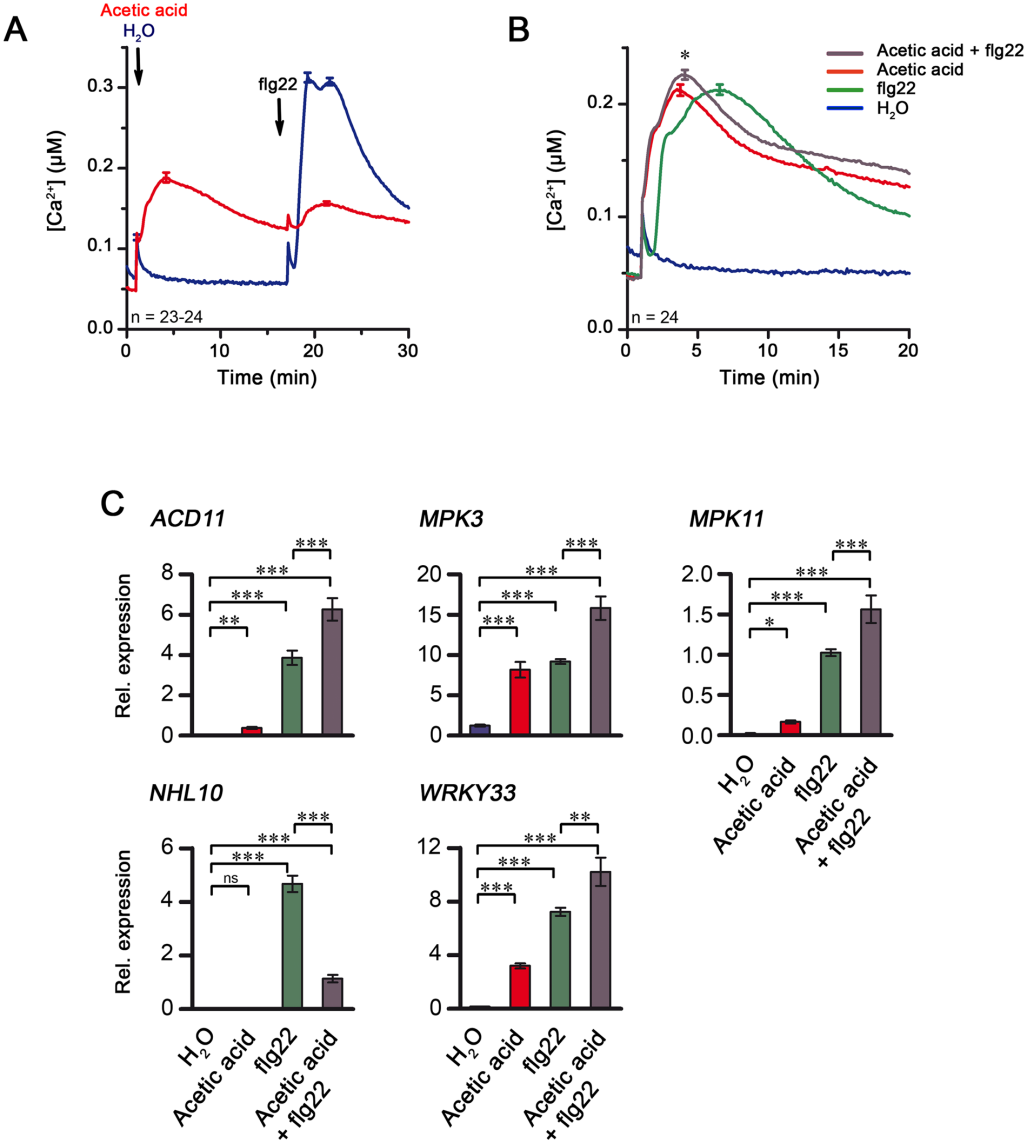
Apolastic acidification induces Ca^2+^ fluxes and alters the response of Arabidopsis to flg22. Pre-treatment of seedlings with low concentrations of acetic acid suppresses the induction of Ca^2+^ flux by flg22, whereas simultaneous application of both stimulants enhances Ca^2+^ response and defense gene expression in comparison to the single treatments. (**A**) Seedlings were treated with water or 0.003% acetic acid for approximately 15 min prior to elicitation with 1 µM flg22. Experiments were performed twice and the curves display the [Ca^2+^]_cyt_ values pooled from the two experiments. Error bars represent standard error of the mean. (**B**) Elicitation of pMAQ2 seedlings was performed with water, an aqueous solution containing 10 nM flg22, 0.003% acetic acid or 0.003% acetic acid containing 10 nM flg22. [Ca^2+^]_cyt_ curves are pooled data from two independent experiments. Error bars represent standard error of the mean. *Significant difference (P < 0.05) according to Two-way ANOVA with Bonferroni post-test. (**C**) Pools of seedlings were elicited with water, an aqueous solution containing 10 nM flg22, 0.006% acetic acid or 0.006% acetic acid containing 10 nM flg22 for 1 hour. Expression of defense genes relative to *PP2AA3* was determined in two independent experiments with 4 seedling pools per treatment and 2 technical replicates per pool. Combined data of both experiments were log-transformed prior to One-way ANOVA with Bonferroni multiple comparison tests for selected columns, as indicated. *,**,***significant difference (P < 0.05, 0.01, 0.001, respectively), ns: not significant.

Hence, acidification by the γ-Glu-Leu solution may explain the observed induced responses. To validate this, we used a dilute acidified solution in place of γ-Glu-Leu. Here, water was acidified with acetic acid to reach the same pH as aqueous γ-Glu-Leu (i.e. ~3.9). Adding this dilute acetic acid solution to Arabidopsis seedlings indeed induced elevations in [Ca^2+^]_cyt_ and “suppressed” the Ca^2+^ rise induced by a subsequent flg22 treatment (Fig. 6A). It also mimicked the enhancement of flg22-induced [Ca^2+^]_cyt_ elevation (Fig. 6B) that was seen with γ-Glu-Leu (c.f. Fig. 4A). Furthermore, co-treatment with acetic acid enhanced the flg22-induced expression levels of *ACD11-like*, *MPK3*, *MPK11* and *WRKY33* (Fig. 6C). However, note that unlike γ-Glu-Leu, addition of dilute acetic acid alone also weakly induced expression of most of these genes, so that the boosted gene expression are additive effects of the co-treatment. Taken together, we could recapitulate the biological effects of γ-Glu-Leu by mimicking apoplastic acidification.

## Discussion

In this work, an extract prepared from *P*. *infestans* mycelium induces Ca^2+^ flux, a typical early defense response, in *A*. *thaliana* seedlings. The C18-pre-fractionated extract contained relatively few compounds and mass spectrometry analysis based on authentic standards revealed one of the components as the dipeptide γ-Glu-Leu. Notably, low-abundance compounds as well as charged molecules cannot be covered by our approach (C18 cartridge and QToF), which should rather be measured with a triple quadrupole mass spectrometer using multi-targeted methods (for low-abundance molecules) or be purified using an ion exchanger cartridge (for charged molecules). For the unknown compounds, CID-MS and H/D exchange chromatography experiments failed to gain further structural hints on those components. In order to elucidate these in future analyses, orthogonal analytical technologies (e.g. GC/MS) or preparative approaches (preparative LC coupled to elemental analysis or NMR analysis) will be required.

Our original goal was to isolate novel *P*. *infestans* MAMPs and use the aequorin-based Ca^2+^ measurements to subsequently identify mutants of the corresponding PRRs. The finding that γ-Glu-Leu elicited Ca^2+^ elevations in *A*. *thaliana* seedlings was initially promising since γ-Glu-Leu-related substances are known to also induce an intracellular [Ca^2+^] increase in animal tissues^53^. Amplitude of [Ca^2+^]_cyt_ increase and the kinetics of the recovery phase were dose-dependent, which is in accordance with various other stresses such as application of hydrogen peroxide^62^, flg22^63,64^ or AtPep1^63^. γ-Glu-Leu also had additive effects on induction of selected flg22-responsive genes. However, γ-Glu-Leu did not (strongly) induce MAPK phosphorylation or ROS accumulation. These observations are reminiscent of recent studies involving cellulose-derived cellobiose fragments acting as a DAMP. Cellobiose weakly activated MPK6 but does not trigger production of ROS or callose deposition; however, it synergistically enhances Arabidopsis defenses in co-treatment with flg22^65^. We also tested if pre-treatment with γ-Glu-Leu would induce resistance to subsequent bacterial infection (as has been shown for other MAMPs, e.g. flg22) but this was not the case (not shown). Thus, γ-Glu-Leu is not a MAMP.

During our analysis, it became eventually clear that all the effects induced by γ-Glu-Leu can be attributed to acidification (of presumably the apoplast and eventually the cytosol). We could abrogate the biological effects of γ-Glu-Leu by buffering the pH exogenously and more importantly, mimicked these effects by applying weak acids like acetic acid (Fig. 6). We had also noticed that in treatments with high concentrations of γ-Glu-Leu, the rate of [Ca^2+^]_cyt_ recovery to resting level was decreased and an apparent second rise in [Ca^2+^]_cyt_, resembling the beginning of some form of Ca^2+^ oscillation, appeared (Figs 3A and S3.). In hindsight, both phenomena may be explainable by acidification. The post-elicitation Ca^2+^ recovery phase relies on both membrane-localized Ca^2+^ pumps and antiporters^17^ to transport Ca^2+^ out of the cytosol. ATPase activity of the pumps and antiporters would be affected by altered proton gradient caused by the acidification in a dose-dependent manner.

The additive effect of γ-Glu-Leu on flg22 induction on some of the analyzed genes was also abolished by buffering the dipeptide solution. Only *WRKY33* retained the boosting effect of γ-Glu-Leu (Fig. 4D). *WRKY33* is one of the upregulated genes found in transcriptome of plants exposed to acidic apoplastic pH^66^. It is possible that the buffering by MES may be incomplete and remnant regional pH changes do occur, which is sufficient to weakly boost flg22-induced expression of acidification-responsive genes such as *WRKY33*. Interestingly, in that report on apoplastic acidification, the transcriptome of plants exposed to low external pH was found to globally cluster with transcriptomes of plants treated with jasmonate, auxin (IAA) or salicylic acid, which are all substances that may lower external pH if not sufficiently buffered. Also clustered to the low pH dataset were transcriptomes of flg22 treatment (1 hpi) and *P*. *infestans* infection (6 and 12 hpi). For the latter, one may speculate that the pH effect of γ-Glu-Leu present on the mycelium may have shaped the *P*. *infestans* infection transcriptome. For flg22, the correlation between low pH and flg22 response is actually in agreement with our observation that the normal Ca^2+^ response to flg22 is dampened when flg22 is MES-buffered (Fig. S9A). This highlights the possibility that for many of the reported studies, and particularly for flg22 treatment, acidification may also contribute to the downstream responses if non-buffered conditions were employed. MapMan analysis of the low pH-regulated genes further revealed an enrichment for “Ca^2+^ regulation” elements^66^ in their promoters, including the “CGCG” core, a promoter element known to be targeted by calcium-regulated CAMTA transcription factors^67^. CAMTA activity or other physiological changes induced by acidification may explain the observed enhancement of gene expression. Thus, this independent observation linking low pH and activation of calcium-mediated response corroborates our observations on the acidification-induced Ca^2+^ response and gene induction through γ-Glu-Leu or weak acids like acetic acid.

While γ-Glu-Leu was originally isolated from *P*. *infestans* mycelium, its physiological function is unknown. In fact, the roles of most naturally occurring dipeptides are unknown; this includes the many dipeptides found in Arabidopsis root exudates^68–70^. Here, it is noteworthy that in the food industry, γ-Glu-Leu belongs to the so-called *Kokumi* taste-enhancing dipeptide family that is found, among others, in beans and mature cheese^71,72^. These *Kokumi* γ-glutamyl peptides act as agonists for the animal extracellular calcium sensing receptor, CaSR^54^ and induce an increase in intracellular [Ca^2+^] in a subset of the CaSR-expressing taste-bud cells within mouse lingual epithelia tissue^53^. Thus, one may also hypothesize that the Glu moiety of γ-Glu-Leu may directly engage analogous calcium-permeating channels of plants such as the ionotropic glutamate receptor-like channels (iGluR)^73,74^, which have been implicated in Ca^2+^ fluxes induced by flg22, elf18 and chitin^75^. While Glu is well established as a second messenger that can mobilize Ca^2+^ through iGluRs, it should be noted that most studies in plants required millimolar concentrations of Glu to elicit [Ca^2+^]_cyt_ changes^76,77^. The recent work on Glu as a systemic wound signal used 100 mM Glu to induce [Ca^2+^]_cyt_ changes and determined apoplastic Glu to reach 50 mM at the damage sites. However, such high concentrations of Glu will certainly involve cellular acidification. Indeed, comparing MES-buffered (or sodium salt of Glu) to unbuffered Glu revealed that Glu-induced Ca^2+^ fluxes may be largely overestimated if pH is not taken into consideration (Fig. S7).

As mentioned above, we initially speculated that the acidic Glu moiety within γ-Glu-Leu would be responsible for the acidification-induced Ca^2+^ response. However, several other dipeptides lacking acidic side chains also induced Ca^2+^ elevation at high concentrations, which is eliminated with MES buffering (Fig. S8), suggesting contribution coming from the carboxyl termini of the dipeptide. Thus, this pH effect may apply to many more peptide elicitors. In this context, we also observed that MES attenuated the Ca^2+^ response induced by flg22 and elf18 but not AtPep1 or chitin (Fig. S9). Compared to flg22 and elf18, AtPep1 is more basic, with one third of the sequence comprising of basic amino acids (lysine, arginine or histidine), which can titrate out the acidic properties of the C-terminus. During the manuscript review of our work, a report appeared showing similar results where flg22- and elf18-induced Ca^2+^ fluxes were reduced by external buffering at pH 5.5^78^. Note additionally that MES did not reduce but rather boosted the Ca^2+^ response of chitin treatment (Fig. S9E). The reason for this boost is unknown at this stage but more importantly, this observation also implied that for all the other elicitors, reduction of Ca^2+^ response when buffered with MES is not simply a counteraction of the plant’s extracellular alkalinization response (Otherwise, the chitin response should also be dampened by MES buffering). Taken together, our current study validates a known but often overlooked fact that apoplastic acidification can induce a rise in [Ca^2+^]_cyt_^79^.

## Conclusions

γ-Glu-Leu is found in *P*. *infestans* mycelium extract and can induce Ca^2+^ flux in Arabidopsis seedlings. Although it can act as a direct agonist of calcium sensing receptor in animal systems, the mechanistic of its Ca^2+^-mobilizing action is unknown in plants. More importantly, all of its elicitor-like properties in plants appear to be due to acidification (presumably apoplastic), and, thus, suggest that γ-Glu-Leu is not a MAMP. Nevertheless, its presence during infection may still contribute to signaling processes that are sensitive to pH alterations. Notably, pH also contributes to the Ca^2+^ signature of established peptide MAMPs such as flg22 or excitatory amino acids such as glutamate. Overall, our work serves as a cautionary reminder that in studies of defense response and (especially?) where plant Ca^2+^ flux measurements are concerned, control of pH is crucial but this is, unfortunately, not always true in the literature.

## Materials and Methods

### Plant material

The Arabidopsis line pMAQ2 was used in all experiments. pMAQ2 expresses cytosolic p35S-apoaequorin in a Col-0 background^80^. Seeds were surface-sterilized in 12-well plates (~20 seeds per well) and stratified at 4 °C for 3–5 days in 2 ml liquid MS medium per well (half strength MS, 0.25% sucrose, 1 mM MES, pH 5.7). Seedlings grew under long day conditions (16 h light, 8 h dark) at 20–22 °C for 7–10 days.

### *P*. *infestans* mycelium extract

*P*. *infestans* isolate CRA208m2^81^ was cultivated in 20 ml (per 100 ml Erlenmeyer flask) of liquid oat bean medium (1.7% [w/v] beanmeal, 0.85% [w/v] oatmeal, 0.425% [w/v] sucrose) in the dark at 18 °C. After 19–22 days, mycelium mats were harvested and thoroughly washed with water. Remaining water was filtered through a nylon mesh placed in a Buchner funnel. For extraction, the protocol of Monjil *et al*.^52^ was modified. Mycelium was ground in liquid nitrogen with a Retsch mill (Retsch GmbH, Haan, Germany) and subsequently homogenized in methanol (1 ml methanol/1 g mycelium) for 2 min using a Polytron (Kinematica AG, Luzern, Switzerland). After centrifugation (30 min, 4 °C, 3000 × g) 1 ml aliquots of the supernatant were evaporated in a SpeedVac (Thermo Fisher Scientific). Pellets were resolved in 1 ml water and combined. To remove salts, extract was loaded onto a Chromabond C_18_ silica column (Chromabond Flash FM 70/10 C_18_ ec, Macherey-Nagel, Düren, Germany), which was treated with methanol prior to use. The flowthrough fraction was discarded and extract components eluted with water (volume equivalent to volume of loaded extract). Thus, 1 ml of extract derived from 100 mg of mycelium. Mycelium and extract were stored at −20 °C.

### Elicitors

Flg22, elf18 and AtPep1^1,2,8^ were synthesized on a ResPep SL peptide synthesizer (Intavis Bioanalytical Instruments). Shrimp shells (Sigma-Aldrich) were used for chitin treatment. Stock solutions of the peptides (1 mM) and shrimp shell chitin (100 mg shrimp shell/ml) were prepared with water, dilutions of the stocks with water, 5 mM MES buffer (pH 6.0) or acetic acid (at the indicated concentrations to attain pH ~3.9, the pH of aqueous γ-Glu-Leu).

### Dipeptides

The dipeptides γ-Glu-Leu and α-Leu-Glu were purchased from Bachem. Leu-Phe, Leu-Ile, Phe-Leu, Tyr-Ile and Ile-Ile were kindly provided by Christoph Böttcher (*Julius-Kühn-Institut*, Berlin). γ-Glu-Ile was synthesized as described in the following. For peptide coupling, protected glutamic acid (303 mg, 1.0 mmol), HOBt (149 mg, 1.1 mmol) and EDC (210 mg, 1.1 mmol) were suspended in dry CH_2_Cl_2_ (10 ml) and stirred at 0 °C for 15 min. Isoleucine t-butyl ester hydrochloride (224 mg, 1.0 mmol) was added, then DIPEA (0.21 ml, 1.2 mmol) was syringed in one portion and the resulting solution was stirred at room temperature overnight (~12 h). The reaction mixture was diluted with 100 ml EtOAc, transferred to a separation funnel and sequentially washed with 0.5 M aqueous citric acid (2 × 50 mL), saturated aqueous NaHCO_3_ (2 × 50 ml) and brine (1 × 30 ml). The organic phase was dried over anhydrous Na_2_SO_4_, filtered and concentrated under reduced pressure. To remove the Boc/OtBu protection groups, the crude peptide was exposed to high vacuum for 1 h before dissolving it in a mixture 3:1 DCM/TFA (5 ml). Pressure from gas evolution generated during the dissolving process was regularly relieved by opening the reaction flask. After 3 h, no starting material was detected by thin layer chromatography and ESI-MS. The volatiles were fully removed under reduced pressure and the resulting thick oily residue was dried at high vacuum for 2 h. Boc-γ-Glu(OtBu)-Ile-OtBu was recovered at a yield of 81% (Rf = 0.73; DCM/MeOH 20:1).

γ-Glu-Leu, α-Leu-Glu and γ-Glu-Ile were dissolved in water or 5 mM MES buffer (pH 6.0). All other dipeptides were dissolved in water (as a 100 mM stock solution), and diluted accordingly with water or 5 mM MES (pH 6.0).

### [Ca^2+^]_cyt_ measurements

Aequorin luminescence measurements were performed as whole seedling assays with individual seedlings (7–10 days old) in 96-well microplates^82^. Ca^2+^ concentrations were calculated as described in Rentel and Knight^83^.

### Gene expression analysis

For gene expression analysis, pools of 10 day old seedlings in 12-well plates were elicited as indicated in figure legends. RNA isolation, reverse transcription and estimation of relative gene expression (relative to *PROTEIN PHOSPHATASE 2A SUBUNIT A3*, *PP2AA3*) were performed as described in Maldonado-Bonilla *et al*.^84^. The primers used are listed in Table S1. Using additional qPCR reference genes (*UBC9* and *UBC21*), the *PP2AA3* gene was tested to be non-responsive to γ-Glu-Leu or apoplastic acidification (Fig. S10) and hence suitable for normalization of gene expression reported in this work.

### Mass spectrometry

Mycelium extracts (20-fold concentrated) were measured on an Acquity UPLC system (Waters, www.waters.com) coupled to a MicrOTOF–Q I hybrid quadrupole time-of-flight mass spectrometer. For this purpose, samples were ionized at positive ESI(+) and negative ESI(−) ionization using an Apollo II electrospray ion source (Bruker Daltonics, www.bruker.com). All mass spectra were acquired in centroid mode and recalibrated on the basis of lithium formate cluster ions. DataAnalysis 4.2 (Bruker Daltonics) was used for the generation of extracted ion chromatograms, deconvolution of mass spectra and calculation of elemental compositions. γ-Glu-Leu was quantified using Quant Analysis 4.2.

### Statistical analysis

Calculation of the EC_50_ value of γ-Glu-Leu and statistical analyses were performed with the software GraphPad Prism 5.0. Details of the statistical analysis are specified in figure legends.

## Supplementary information

Supplementary Info- Westphal et al

## Data Availability

All data generated or analysed during this study are included in this published article (and its Supplementary Information files). Other materials and raw data are available upon request or deposited on data repository sites.

## References

[1] Gómez-Gómez L, Felix G, Boller T (1999). A single locus determines sensitivity to bacterial flagellin in Arabidopsis thaliana. Plant J.

[2] Kunze G (2004). The N terminus of bacterial elongation factor Tu elicits innate immunity in Arabidopsis plants. Plant Cell.

[3] Zipfel C (2006). Perception of the bacterial PAMP EF-Tu by the receptor EFR restricts Agrobacterium-mediated transformation. Cell.

[4] Miya A (2007). CERK1, a LysM receptor kinase, is essential for chitin elicitor signaling in Arabidopsis. Proc Natl Acad Sci USA.

[5] Petutschnig EK, Jones AM, Serazetdinova L, Lipka U, Lipka V (2010). The LysM-RLK CERK1 is a major chitin binding protein in *Arabidopsis thaliana* and subject to chitin-induced phosphorylation. J Biol Chem.

[6] Wan J (2008). A LysM receptor-like kinase plays a critical role in chitin signaling and fungal resistance in Arabidopsis. Plant Cell.

[7] Cao, Y. *et al*. The kinase LYK5 is a major chitin receptor in Arabidopsis and forms a chitin-induced complex with related kinase CERK1. *Elife* **3**, 10.7554/eLife.03766 (2014). 10.7554/eLife.03766 PMC4356144 25340959

[8] Huffaker A, Pearce G, Ryan CA (2006). An endogenous peptide signal in Arabidopsis activates components of the innate immune response. Proceedings of the National Academy of Sciences.

[9] Krol E (2010). Perception of the Arabidopsis danger signal peptide 1 involves the pattern recognition receptor AtPEPR1 and its close homologue AtPEPR2. J Biol Chem.

[10] Yamaguchi Y, Huffaker A, Bryan AC, Tax FE, Ryan CA (2010). PEPR2 Is a Second Receptor for the Pep1 and Pep2 Peptides and Contributes to Defense Responses in Arabidopsis. Plant Cell.

[11] Couto D, Zipfel C (2016). Regulation of pattern recognition receptor signalling in plants. Nat Rev Immunol.

[12] Knogge, W., Lee, J., Rosahl, S. & Scheel, D. In *Plant Relationships* Vol. V *The* Mycota (ed. Holger B. Deising) 337–361 (Springer Berlin Heidelberg, 2009).

[13] Lee, J., Eschen-Lippold, L., Lassowskat, I., Bottcher, C. & Scheel, D. Cellular reprogramming through mitogen-activated protein kinases. *Frontiers in Plant Science* **6**, 10.3389/fpls.2015.00940 (2015). 10.3389/fpls.2015.00940 PMC4625042 26579181

[14] Li B, Meng X, Shan L, He P (2016). Transcriptional Regulation of Pattern-Triggered Immunity in Plants. Cell Host Microbe.

[15] Vidhyasekaran, P. *PAMP Signals in Plant Innate Immunity: signal perception and transduction*. XVII, 442 (SPRINGER netherlands, 2016).

[16] McAinsh MR, Brownlee C, Hetherington AM (1997). Calcium ions as second messengers in guard cell signal transduction. Physiol Plantarum.

[17] Seybold H (2014). Ca2+ signalling in plant immune response: from pattern recognition receptors to Ca2+ decoding mechanisms. New Phytologist.

[18] Blume B, Nürnberger T, Nass N, Scheel D (2000). Receptor-mediated increase in cytoplasmic free calcium required for activation of pathogen defense in parsley. Plant Cell.

[19] Ranf S (2014). Microbe-associated molecular pattern-induced calcium signaling requires the receptor-like cytoplasmic kinases, PBL1 and BIK1. Bmc Plant Biology.

[20] Ranf S (2012). Defense-Related Calcium Signaling Mutants Uncovered via a Quantitative High-Throughput Screen in Arabidopsis thaliana. Molecular Plant.

[21] Farid A (2013). Specialized roles of the conserved subunit OST3/6 of the oligosaccharyltransferase complex in innate immunity and tolerance to abiotic stresses. Plant Physiol.

[22] Trempel F (2016). Altered glycosylation of exported proteins, including surface immune receptors, compromises calcium and downstream signaling responses to microbe-associated molecular patterns in Arabidopsis thaliana. Bmc Plant Biology.

[23] Choi J (2014). Identification of a plant receptor for extracellular ATP. Science.

[24] Ranf S (2015). A lectin S-domain receptor kinase mediates lipopolysaccharide sensing in Arabidopsis thaliana. Nature Immunology.

[25] Hahlbrock K (1995). Oligopeptide elicitor-mediated defense gene activation in cultured parsley cells. Proc Natl Acad Sci USA.

[26] Gust AA (2007). Bacteria-derived peptidoglycans constitute pathogen-associated molecular patterns triggering innate immunity in Arabidopsis. Journal of Biological Chemistry.

[27] Moroz N (2017). Extracellular Alkalinization as a Defense Response in Potato Cells. Front Plant Sci.

[28] Mathieu Y, Jouanneau JP, Thomine S, Lapous D, Guern J (1994). Cytosolic protons as secondary messengers in elicitor-induced defence responses. Biochem Soc Symp.

[29] Roos W, Evers S, Hieke M, Tschope M, Schumann B (1998). Shifts of intracellular pH distribution as a part of the signal mechanism leading to the elicitation of benzophenanthridine alkaloids. Phytoalexin biosynthesis in cultured cells of eschscholtzia californica. Plant Physiol.

[30] Michard E, Simon AA, Tavares B, Wudick MM, Feijo JA (2017). Signaling with Ions: The Keystone for Apical Cell Growth and Morphogenesis in Pollen Tubes. Plant Physiol.

[31] Herrmann A, Felle HH (1995). Tip Growth in Root Hair-Cells of Sinapis-Alba L - Significance of Internal and External Ca2+ and Ph. New Phytologist.

[32] Monshausen GB, Bibikova TN, Messerli MA, Shi C, Gilroy S (2007). Oscillations in extracellular pH and reactive oxygen species modulate tip growth of Arabidopsis root hairs. Proc Natl Acad Sci USA.

[33] Monshausen GB, Messerli MA, Gilroy S (2008). Imaging of the Yellow Cameleon 3.6 indicator reveals that elevations in cytosolic Ca2+ follow oscillating increases in growth in root hairs of Arabidopsis. Plant Physiol.

[34] Monshausen GB, Miller ND, Murphy AS, Gilroy S (2011). Dynamics of auxin-dependent Ca2+ and pH signaling in root growth revealed by integrating high-resolution imaging with automated computer vision-based analysis. Plant J.

[35] Dindas J (2018). AUX1-mediated root hair auxin influx governs SCF(TIR1/AFB)-type Ca(2+) signaling. Nature communications.

[36] Monshausen GB, Bibikova TN, Weisenseel MH, Gilroy S (2009). Ca2+ regulates reactive oxygen species production and pH during mechanosensing in Arabidopsis roots. Plant Cell.

[37] Behera S (2018). Cellular Ca(2+) Signals Generate Defined pH Signatures in Plants. Plant Cell.

[38] Sogin ML, Silberman JD (1998). Evolution of the protists and protistan parasites from the perspective of molecular systematics. Int J Parasitol.

[39] Huitema E, Vleeshouwers VG, Francis DM, Kamoun S (2003). Active defence responses associated with non-host resistance of Arabidopsis thaliana to the oomycete pathogen Phytophthora infestans. Mol Plant Pathol.

[40] Lipka V (2005). Pre- and postinvasion defenses both contribute to nonhost resistance in Arabidopsis. Science.

[41] Fawke S, Doumane M, Schornack S (2015). Oomycete Interactions with Plants: Infection Strategies and Resistance Principles. Microbiology and Molecular Biology Reviews.

[42] Raaymakers, T. M. & Van den Ackerveken, G. Extracellular Recognition of Oomycetes during Biotrophic Infection of Plants. *Frontiers in Plant Science* **7**, 10.3389/fpls.2016.00906 (2016). 10.3389/fpls.2016.00906 PMC4915311 27446136

[43] Gaulin E (2006). Cellulose Binding Domains of a Phytophthora Cell Wall Protein Are Novel Pathogen-Associated Molecular Patterns. The Plant Cell.

[44] Séjalon-Delmas N (1997). Purification, Elicitor Activity, and Cell Wall Localization of a Glycoprotein from Phytophthora parasitica var. nicotianae, a Fungal Pathogen of Tobacco. Phytopathology.

[45] Khatib M, Lafitte C, Esquerre-Tugaye MT, Bottin A, Rickauer M (2004). The CBEL elicitor of Phytophthora parasitica var. nicotianae activates defence in Arabidopsis thaliana via three different signalling pathways. New Phytologist.

[46] Dumas B, Bottin A, Gaulin E, Esquerre-Tugaye MT (2008). Cellulose-binding domains: cellulose associated-defensive sensing partners?. Trends Plant Sci.

[47] Gijzen M, Nurnberger T (2006). Nep1-like proteins from plant pathogens: recruitment and diversification of the NPP1 domain across taxa. Phytochemistry.

[48] Oome S (2014). & Van den Ackerveken, G. Comparative and functional analysis of the widely occurring family of Nep1-like proteins. Mol Plant Microbe Interact.

[49] Bohm H, Albert I, Fan L, Reinhard A, Nurnberger T (2014). Immune receptor complexes at the plant cell surface. Current Opinion in Plant Biology.

[50] Bi G (2014). Arabidopsis thaliana receptor-like protein AtRLP23 associates with the receptor-like kinase AtSOBIR1. Plant Signaling & Behavior.

[51] Albert I (2015). An RLP23–SOBIR1–BAK1 complex mediates NLP-triggered immunity. *Nature*. Plants.

[52] Monjil MS, Wada S, Takemoto D, Kawakita K (2013). Non-host resistance activities of *Arabidopsis thaliana* induced by methanol extract of mycelia from *Phytophthora infestans*. International Journal of Biosciences and Biotechnology.

[53] Maruyama Y, Yasuda R, Kuroda M, Eto Y (2012). Kokumi substances, enhancers of basic tastes, induce responses in calcium-sensing receptor expressing taste cells. PLoS One.

[54] Ohsu T (2010). Involvement of the calcium-sensing receptor in human taste perception. J Biol Chem.

[55] Fauth M (1998). Cutin monomers and surface wax constituents elicit H2O2 in conditioned cucumber hypocotyl segments and enhance the activity of other H2O2 elicitors. Plant Physiol.

[56] Aslam SN (2009). Microbe-associated molecular pattern (MAMP) signatures, synergy, size and charge: influences on perception or mobility and host defence responses. Mol Plant Pathol.

[57] Flury P, Klauser D, Schulze B, Boller T, Bartels S (2013). The anticipation of danger: microbe-associated molecular pattern perception enhances AtPep-triggered oxidative burst. Plant Physiol.

[58] Bethke G (2012). Activation of the Arabidopsis thaliana mitogen-activated protein kinase MPK11 by the flagellin-derived elicitor peptide, flg22. Mol Plant Microbe Interact.

[59] Boudsocq M (2010). Differential innate immune signalling via Ca^2+^ sensor protein kinases. Nature.

[60] Pecher P (2014). The Arabidopsis thaliana mitogen-activated protein kinases MPK3 and MPK6 target a subclass of “VQ-motif’-containing proteins to regulate immune responses. New Phytologist.

[61] Tena G, Renaudin JP (1998). Cytosolic acidification but not auxin at physiological concentration is an activator of MAP kinases in tobacco cells. Plant J.

[62] Knight H, Trewavas AJ, Knight MR (1997). Calcium signalling in *Arabidopsis thaliana* responding to drought and salinity. Plant J.

[63] Cao, X. Q. *et al*. Biotic and Abiotic Stresses Activate Different Ca2+ Permeable Channels in Arabidopsis. *Frontiers in Plant Science* **8**, 10.3389/fpls.2017.00083 (2017). 10.3389/fpls.2017.00083 PMC5281638 28197161

[64] Jeworutzki E (2010). Early signaling through the Arabidopsis pattern recognition receptors FLS2 and EFR involves Ca2+-associated opening of plasma membrane anion channels. Plant Journal.

[65] de Azevedo Souza, C. *et al*. Cellulose-derived oligomers act as damage-associated molecular patterns and trigger defense-like responses. *Plant Physiology*, 10.1104/pp.16.01680 (2017). 10.1104/pp.16.01680 PMC5373054 28242654

[66] Lager I (2010). Changes in external pH rapidly alter plant gene expression and modulate auxin and elicitor responses. Plant Cell Environ.

[67] Whalley HJ (2011). Transcriptomic analysis reveals calcium regulation of specific promoter motifs in Arabidopsis. Plant Cell.

[68] Moussaieff A (2013). High-resolution metabolic mapping of cell types in plant roots. Proc Natl Acad Sci USA.

[69] Strehmel N, Böttcher C, Schmidt S, Scheel D (2014). Profiling of secondary metabolites in root exudates of *Arabidopsis thaliana*. Phytochemistry.

[70] Strehmel N (2017). Stress-Related Mitogen-Activated Protein Kinases Stimulate the Accumulation of Small Molecules and Proteins in Arabidopsis thaliana Root Exudates. Frontiers in Plant Science.

[71] Shibata M (2017). Isolation and characterization of key contributors to the “kokumi” taste in soybean seeds. Biosci Biotechnol Biochem.

[72] Toelstede S, Dunkel A, Hofmann T (2009). A series of kokumi peptides impart the long-lasting mouthfulness of matured Gouda cheese. J Agric Food Chem.

[73] Meyerhoff O (2005). AtGLR3.4, a glutamate receptor channel-like gene is sensitive to touch and cold. Planta.

[74] Qi Z, Stephens NR, Spalding EP (2006). Calcium Entry Mediated by GLR3.3, an Arabidopsis Glutamate Receptor with a Broad Agonist Profile. Plant Physiology.

[75] Kwaaitaal M, Huisman R, Maintz J, Reinstadler A, Panstruga R (2011). Ionotropic glutamate receptor (iGluR)-like channels mediate MAMP-induced calcium influx in *Arabidopsis thaliana*. Biochem J.

[76] Dennison KL, Spalding EP (2000). Glutamate-Gated Calcium Fluxes in Arabidopsis. Plant Physiology.

[77] Dubos C, Huggins D, Grant GH, Knight MR, Campbell MM (2003). A role for glycine in the gating of plant NMDA-like receptors. Plant J.

[78] Wang YC, Yu M, Shih PY, Wu HY, Lai EM (2018). Stable pH Suppresses Defense Signaling and is the Key to Enhance Agrobacterium-Mediated Transient Expression in Arabidopsis Seedlings. Scientific reports.

[79] Zhu X, Feng Y, Liang G, Liu N, Zhu JK (2013). Aequorin-based luminescence imaging reveals stimulus- and tissue-specific Ca2+ dynamics in Arabidopsis plants. Mol Plant.

[80] Knight MR, Campbell AK, Smith SM, Trewavas AJ (1991). Transgenic plant aequorin reports the effects of touch and cold-shock and elicitors on cytoplasmic calcium. Nature.

[81] Si-Ammour A, Mauch-Mani B, Mauch F (2003). Quantification of induced resistance against Phytophthora species expressing GFP as a vital marker: beta-aminobutyric acid but not BTH protects potato and Arabidopsis from infection. Molecular Plant Pathology.

[82] Trempel F, Ranf S, Scheel D, Lee J (2016). Quantitative analysis of microbe-associated molecular pattern (MAMP)-induced Ca^2+^ transients in plants. Methods Mol Biol.

[83] Rentel MC, Knight MR (2004). Oxidative stress-induced calcium signaling in Arabidopsis. Plant Physiol.

[84] Maldonado-Bonilla LD (2014). The Arabidopsis Tandem Zinc Finger 9 Protein Binds RNA and Mediates Pathogen-Associated Molecular Pattern-Triggered Immune Responses. Plant and Cell Physiology.

