# Supplementary material for: pH effects on plant calcium fluxes: lessons from acidification-mediated calcium elevation induced by the γ-glutamyl-leucine dipeptide identified from Phytophthora infestans
Source: Sci Rep. 2019 Mar 18;9:4733. doi: 10.1038/s41598-019-41276-0 (PMC6426842; doi:10.1038/s41598-019-41276-0)
Supplement: Supplementary file 1 — Supplementary Info- Westphal et al [file 41598_2019_41276_MOESM1_ESM.pdf]

Supporting information

pH effects on plant calcium fluxes: lessons from acidification-mediated calcium elevation induced by the γ-glutamyl-leucine dipeptide identified from *Phytophthora infestans*.

Lore Westphal, Nadine Strehmel, Lennart Eschen-Lippold, Nicole Bauer, Bernhard Westermann, Sabine Rosahl, Dierk Scheel, Justin Lee*

* Corresponding author: jlee@ipb-halle.de

Contains: 11 figures

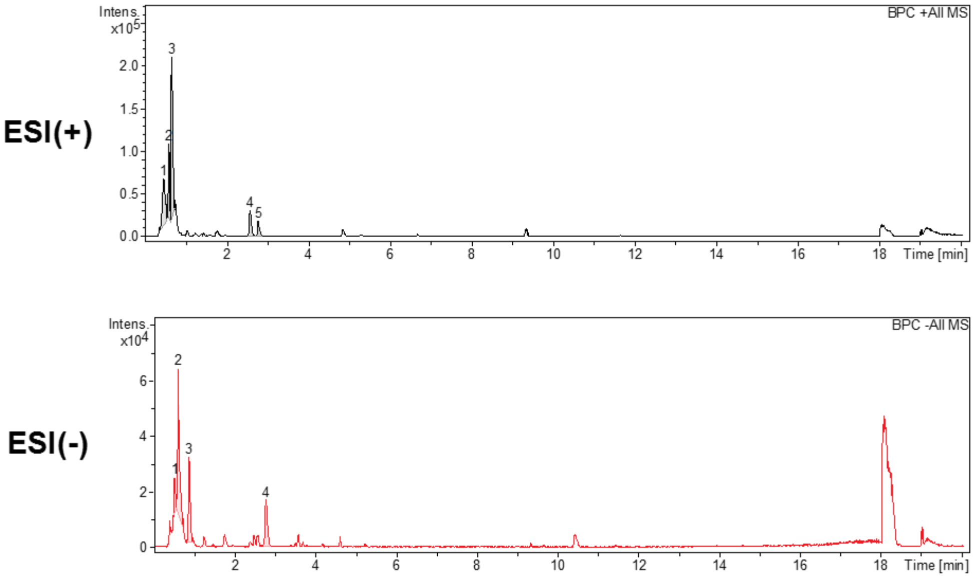

S1 Fig. Representative base peak chromatograms of *P. infestans* medium as recorded by UPLC/ESI(+)-QTOFMS and UPLC/ESI(-)-QTOFMS. ESI(+): 1) trigonelline; 2) $C_9H_{17}NO_4$; 3) tyrosine; 4) γ-Glu-Leu; 5) $C_9H_{10}O_5$ and ESI(-): 1) citric acid; 2) isocitric acid; 3) $C_{14}H_{18}N_2O_6$; 4) $C_{16}H_{22}O_{10}$.

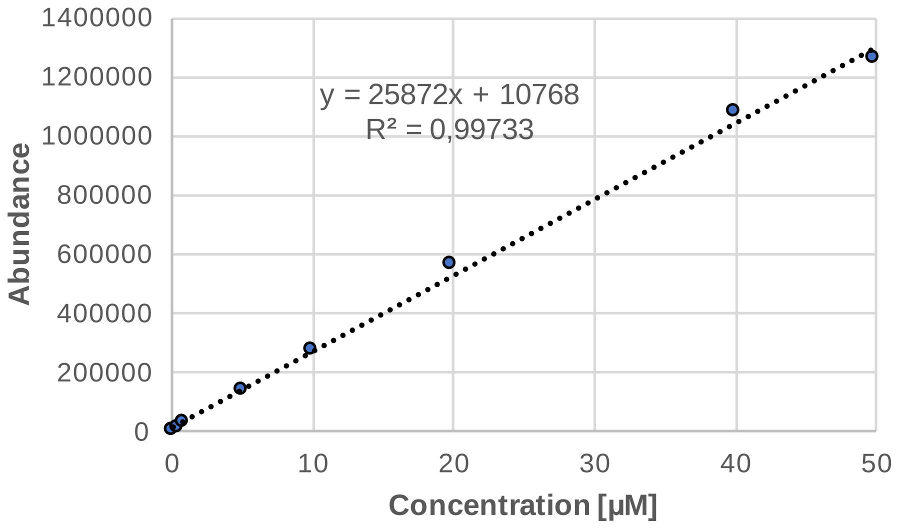

S2 Fig. Quantitation of γ-Glu-Leu in mycelium extract of *P. infestans*.

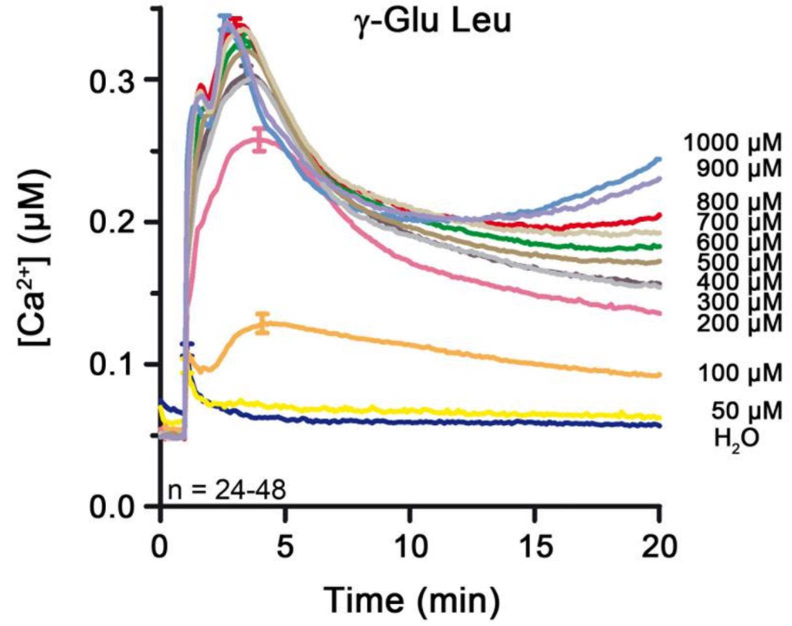

S3 Fig. γ-Glu-Leu induces Ca^{2+} flux in Arabidopsis pMAQ2 in a concentration-dependent manner. Seedlings were treated with water and aqueous solutions containing different concentrations of γ-Glu-Leu, respectively (two to four independent experiments per concentration). Error bars represent standard error of the mean.

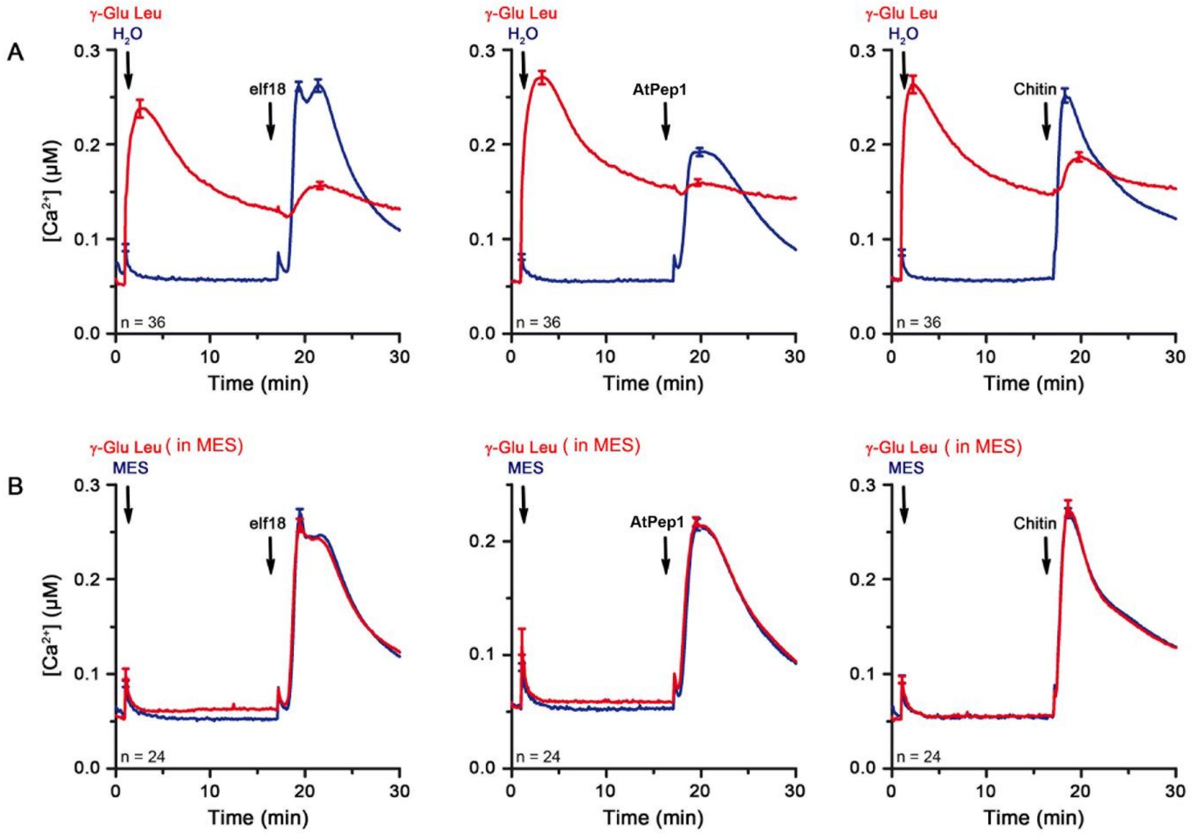

S4 Fig. γ-Glu-Leu desensitizes pMAQ2 seedlings for subsequent treatments with elf18, AtPep1 and chitin when applied as aqueous solution, but not when dissolved in MES buffer (pH 6.0). (A) Arabidopsis pMAQ2 seedlings were treated with water or an aqueous solution containing 500 µM γ-Glu-Leu approximately 15 min before the application of elf18 (1 µM), AtPep1 (1 µM) or chitin (200 µg shrimp shell/ml). (B) Seedlings were treated with MES buffer (pH 6.0) or MES buffer containing 500 µM γ-Glu-Leu (pH 6.0) approximately 15 min before the application of elf18 (1 µM), AtPep1 (1 µM) or chitin (200 µg shrimp shell/ml). Experiments were performed twice and the data of both experiments were pooled. Error bars represent standard error of the mean.

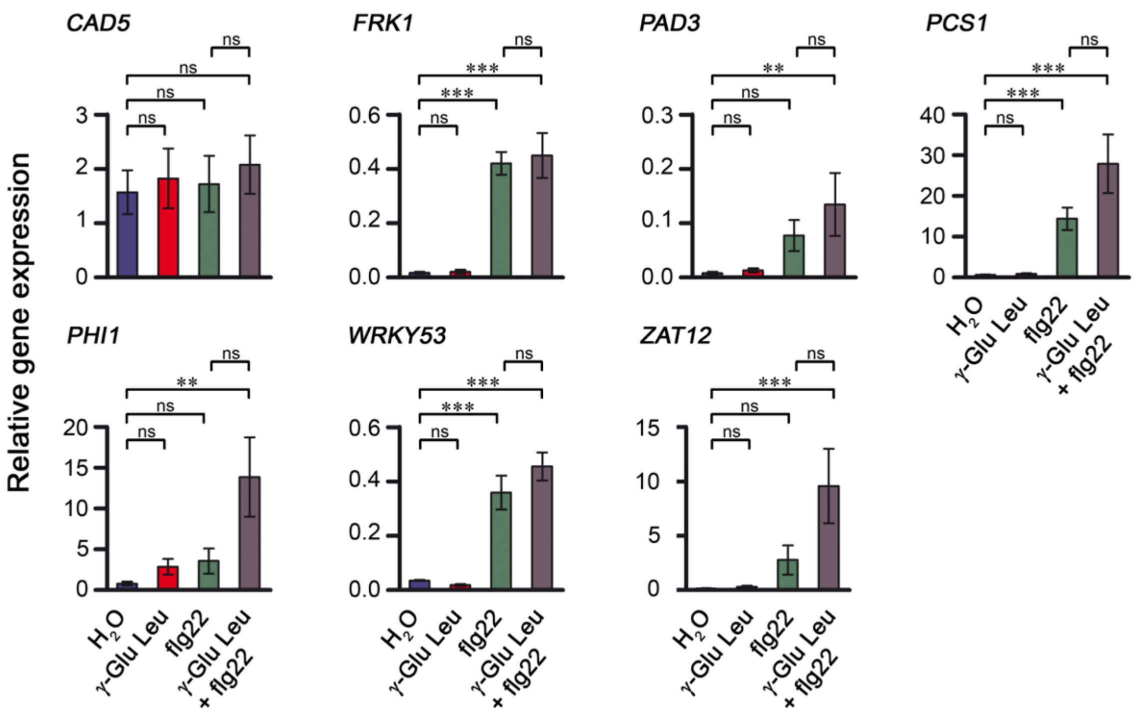

S5 Fig. Co-treatment of Arabidopsis with γ-Glu-Leu and flg22 has no significant influence on the expression of *CAD5*, *FRK1*, *PAD3*, *PCS1*, *PHI1*, *WRKY53* and *ZAT12* in comparison to the flg22 treatment. Pools of pMAQ2 seedlings were treated with water or an aqueous solution containing 10 nM flg22 and/or 500 µM γ-Glu-Leu for 1 hour. Expression of defense genes (relative to *PP2AA3*) was determined in two independent experiments with 3-4 seedling pools per treatment and 2 technical replicates per pool. Combined data of both experiments were log-transformed prior to One-way ANOVA with Bonferroni multiple comparison tests. **, *** = significant difference ($P<0.01$, 0.001, respectively), ns: not significant.

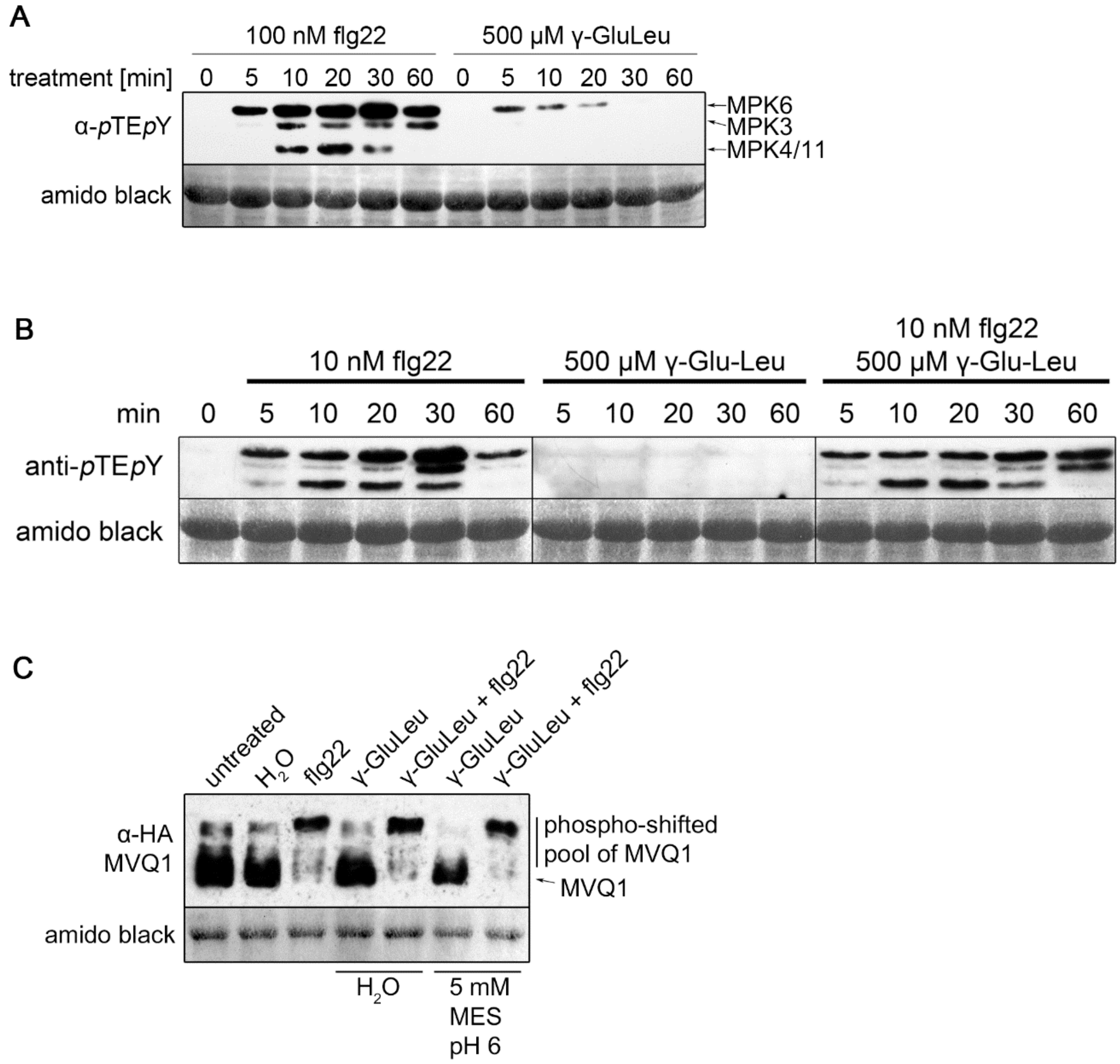

S6 Fig. γ-Glu-Leu does not enhance MAPK activation or MPK6-dependent phosphorylation of MVQ1. (A) pMAQ2 seedlings were grown in half strength MS-media and treated with 100 nM flg22 or 500 µM γ-Glu-Leu for the indicated time points. Protein extracts were subjected to Western blot analysis using an antibody detecting phosphorylated (activated) MAPKs (α-*p*TE*p*Y). (B) Samples were generated as in (A), except with simultaneous co-treatment of 10 nM flg22 and 500 µM γ-Glu-Leu. Note the weak MAPK phosphorylation by γ-Glu-Leu (as seen in A above) is not visible at this exposure of the western blot. (C) Col-0 protoplasts were transformed with an MVQ1-HA construct (Pecher et al. 2014). After 16 h, samples were treated for 1 h with H_2O as a control, 100 nM flg22, 500 µM γ-Glu-Leu or both solutions simultaneously in either water or buffered in MES (pH 6). Amido black staining of the membranes shows equal loading (large subunit of RuBisCO). For data transparency, the original uncropped blot images can be seen in Supplementary Figure S11.

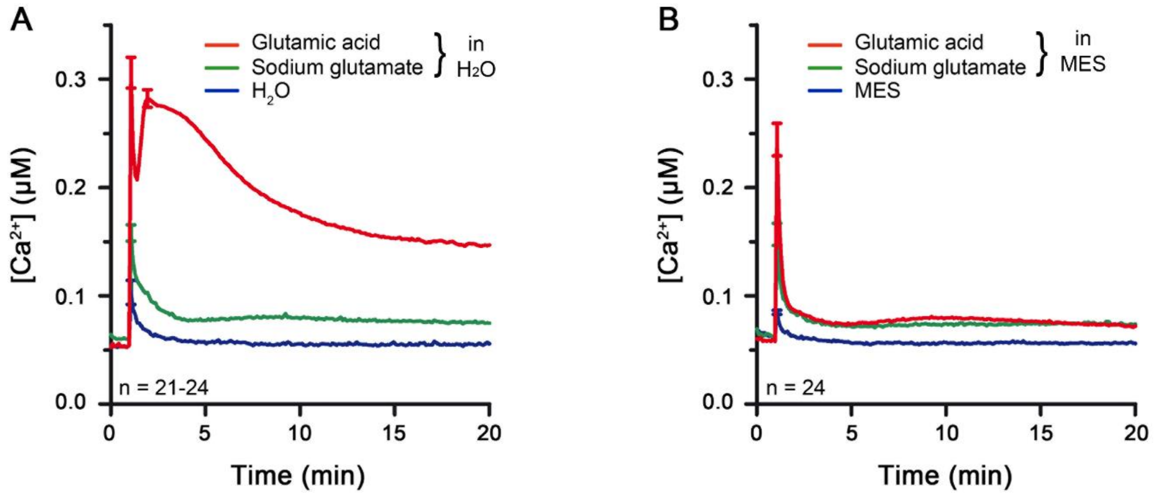

S7 Fig. Unbuffered glutamic acid solution “overinflates” the actual Ca^{2+} response in pMAQ2 seedlings. (A) Arabidopsis pMAQ2 seedlings were treated with water and aqueous solutions containing 1 mM Glutamic acid (as free acid) or as sodium salt. (B) Seedlings were treated with MES (pH 6.0) and MES solutions (pH 6.0) containing 1 mM Glutamic acid or Sodium glutamate. Experiments were performed twice and the data of both experiments were pooled. Error bars represent standard error of the mean.

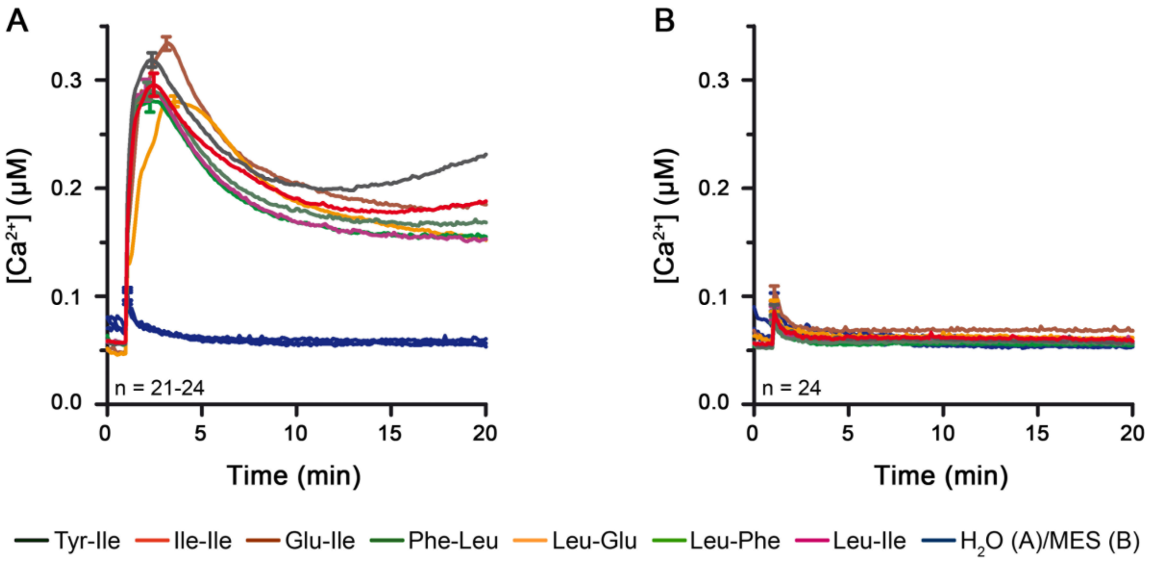

S8 Fig. A variety of different dipeptides induce Ca^{2+} flux in Arabidopsis pMAQ2 as aqueous solution but not when buffered with MES (pH 6.0). (A) Seedlings were treated with water and aqueous solutions containing 500 µM of Tyr-Ile, Ile-Ile, Glu-Ile, Phe-Leu, Leu-Glu, Leu-Phe or Leu-Ile. (B) Seedlings were treated with MES (pH 6.0) and MES solutions containing 500 µM of Tyr-Ile, Ile-Ile, Glu-Ile, Phe-Leu, Leu-Glu, Leu-Phe or Leu-Ile (pH 6.0). $[Ca^{2+}]_{cyt}$ curves are based on pooled data from two independent experiments. Error bars represent standard error of the mean.

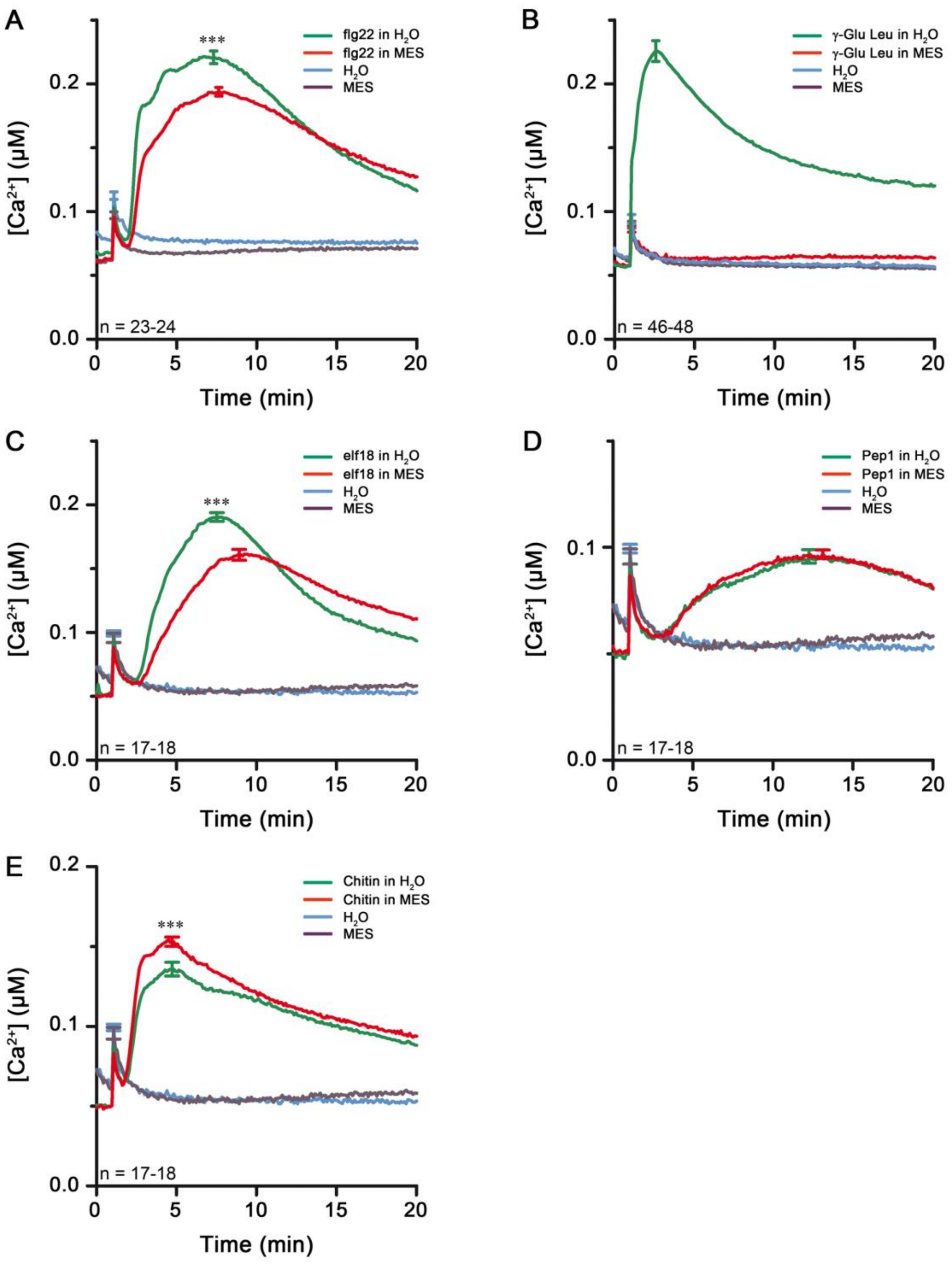

S9 Fig. MES buffer (pH 6.0) diminishes the Ca^{2+} response of Arabidopsis pMAQ2 to flg22 and eliminates the response to γ-Glu-Leu. (A-E) Seedlings were treated with the indicated elicitors dissolved in either water or MES buffer (pH 6.0). Elicitors used were 10 nM flg22 (A), 250 µM γ-Glu-Leu (B), 10 nM elf18 (C), 10 nM AtPep1 (D) and 2 µg shrimp shell/ml (E). Experiments were performed twice (A) four times (B) or three times (C-E) and data were pooled. Error bars represent standard error of the mean. *** = significant difference (P<0.001) according to Two-way ANOVA with Bonferroni post-test. Note that (B) is replotted using the data from Fig 4A and B.

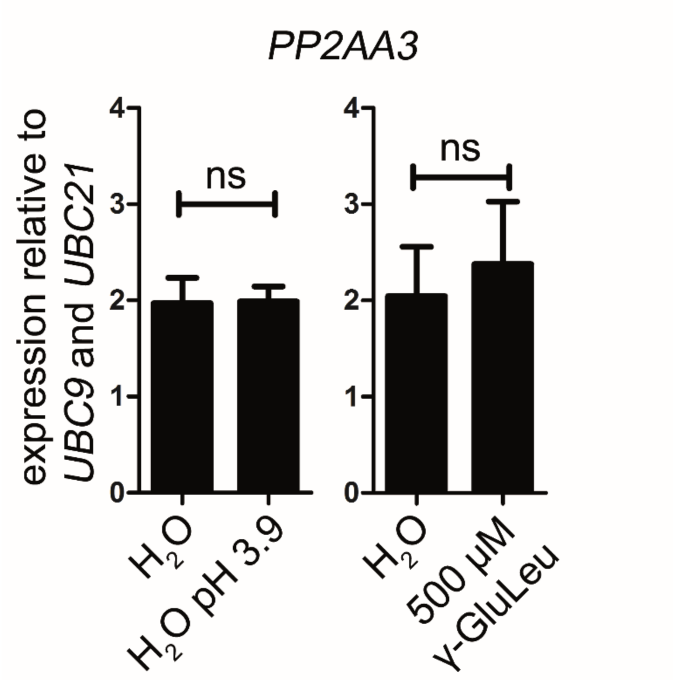

S10 Fig. qRT-PCR analysis of the influence of γ-Glu-Leu or acidification on *PP2A* expression. Pools of pMAQ2 seedlings were elicited with water, water adjusted to pH 3.9, or 500 µM γ-Glu-Leu for 1 hour. Expression of *PP2AA3* relative to *UBIQUITIN CONJUGATING ENZYME9* and *21* (*UBC9*, *UBC21*) was determined in two independent experiments with 4 seedling pools per treatment and 2 technical replicates per pool. Combined data of both experiments were log-transformed prior to two-tailed Mann-Whitney t-tests; ns = no significant difference.

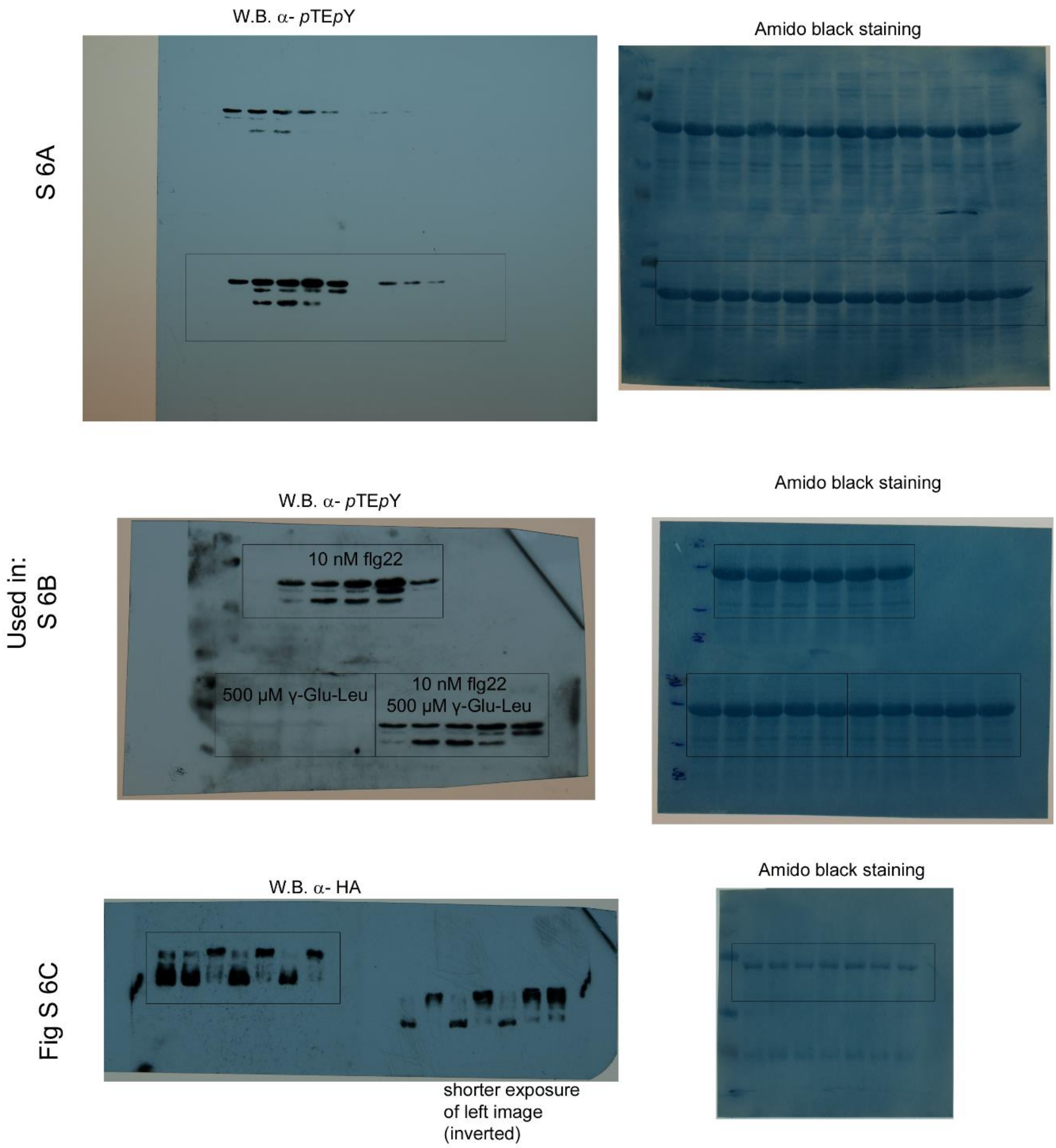

S11 Fig. The original western blots used to assemble Fig. S6. The cropped regions used for Fig S6 are framed with boxes. Experimental details and labelling of lanes are described in legend of Fig S6.

Table S1: Oligonucleotide primers used in qRT-PCR

Primer name	AGI code	sequence
PP2AA3_5	At1G13320	gaccggagccaactaggac
PP2AA3_3		aaaacttggtaacttttccagca
UBC9_5	At4G27960	tcacaatttccaaggtgctgc
UBC9_3		tcatctgggtttggatccgt
UBC21_5	At5G25760	cagtctgtgtgtagagctatcatagcat
UBC21_3		agaagattccctgagtcgcagtt
ACD11-like_5	AT4G39670	gtacatcgccaaggttaagga
ACD11-like_3		tctaggtcaagaatgttgtgtaacg
WRKY33_5	AT2G38470	gggaaacccaaatccaaga
WRKY33_3		gtttcccttcgtaggttgtga
MPK11_5	AT1G01560	gcttctggcatcgtctgtg
MPK11_3		ctcaaagttctcttagcgtcgat
MPK3_5	AT3G45640	tcaagcttcttcgtcatcttga
MPK3_3		tgatttgatgaagatcagtatccatta
NHL10_5	AT2G35980	acgccggacagtctagga
NHL10_3		ccctaagcctgaacttgatctc
PAD3_5	AT3G26830	caccactgatcatctcaaagga
PAD3_3		cggtcattccccatagtgtt
PCS1_5	AT5G44070	acatatcaccgaggtgtatttaagc
PCS1_3		gccaccaataggtgaaaagtg
FRK1_5	AT2G19190	gagactatttggcaggtaaaaggt
FRK1_3		aggaggcttacaaccattgtg
CAD5_3	AT4G34230	tgtctctgcttaagcttgatgg
CAD5_5		tgaagctccccgttatcact
WRKY53_5	AT4G23810	cggaagtccgagaagtgaag
WRKY53_3		tctgaccactttggtaacatcttt
ZAT12_5	AT5G59820	ctttgggaggacacatgagg
ZAT12_3		caaagcgcgtgtaaccaac
PHI1_5	AT1G35140	tggatattaccaagggcctaaa
PHI1_3		ccagcataaccaggatacgaa
